# Standardised comparison of limonene-derived monoterpenes identifies structural determinants of anti-inflammatory activity

**DOI:** 10.1038/s41598-020-64032-1

**Published:** 2020-04-29

**Authors:** Cátia Sousa, Alcino Jorge Leitão, Bruno Miguel Neves, Fernando Judas, Carlos Cavaleiro, Alexandrina Ferreira Mendes

**Affiliations:** 1 Centre for Neuroscience and Cell Biology, University of Coimbra, Coimbra, Portugal; 2 Faculty of Pharmacy, University of Coimbra, Coimbra, Portugal; 3 Centre for Innovative Biomedicine and Biotechnology, University of Coimbra, Coimbra, Portugal; 4 Department of Medical Sciences and Institute of Biomedicine – iBiMED, University of Aveiro, Aveiro, Portugal; 5 Orthopaedics Department and Bone Bank, University and Hospital Centre of Coimbra, Coimbra, Portugal; 6 Chemical Process Engineering and Forest Products Research Centre, Chemical Engineering Department, Faculty of Sciences and Technology, University of Coimbra, Coimbra, Portugal

**Keywords:** Cell biology, Chemical biology, Medical research

## Abstract

Mint species are widely used in traditional and conventional medicine as topical analgesics for osteoarthritic pain and for disorders of the gastrointestinal and respiratory tracts which are all associated with chronic inflammation. To identify the structural determinants of anti-inflammatory activity and potency which are required for chemical optimization towards development of new anti-inflammatory drugs, a selected group of monoterpenes especially abundant in mint species was screened by measuring bacterial lipopolysacharide (LPS)-induced nitric oxide (NO) production in murine macrophages. Nine compounds significantly decreased LPS-induced NO production by more than 30%. IC_50_ values were calculated showing that the order of potency is: (S)-(+)-carvone > (R)-(−)-carvone > (+)-dihydrocarveol > (S)-8-hydroxycarvotanacetone > (R)-8-hydroxycarvotanacetone > (+)-dihydrocarvone > (−)-carveol > (−)-dihydrocarveol > (S)-(-)-pulegone. Considering the carbon numbering relative to the common precursor, limonene, the presence of an oxygenated group at C6 conjugated to a double bond at C1 and an isopropenyl group and S configuration at C4 are the major chemical features relevant for activity and potency. The most potent compound, (S)-(+)-carvone, significantly decreased the expression of NOS2 and IL-1β in macrophages and in a cell model of osteoarthritis using primary human chondrocytes. (S)-(+)-carvone may be efficient in halting inflammation-related diseases, like osteoarthritis.

## Introduction

Inflammation is an orchestrated physiological response elicited by exogenous inducers such as infectious agents, allergens, irritants and toxic compounds, as well as by endogenous triggers released from stressed or damaged tissues/cells^1^. Although aiming at restoring homeostasis, inflammation has the potential to cause tissue damage and perpetuate itself^2^. Likewise, inflammation has been reported as an important component associated with most chronic human diseases, such as rheumatic^3^, metabolic and neurodegenerative diseases and cancer^2,4^. Due to the increased incidence of these diseases in relation with population aging and the lack of efficacy and adverse side effects of currently available anti-inflammatory drugs more directed to acute inflammation, new therapeutic agents are needed to contend chronic inflammation-associated diseases^5–7^.

Natural products are increasingly used for their anti-inflammatory properties and as sources of new anti-inflammatory compounds^5,6^. Among the species most widely used, those of the family Lamiaceae, genus *Mentha* L., commonly designated as mint species, are widely used in traditional^8^ and conventional medicine, especially as essential oils. These are well-known for anti-inflammatory, antimicrobial, carminative, antispasmodic and analgesic properties. Among several chemical classes identified in mint essential oils, monoterpenes belonging to the limonene synthase pathway, such as menthol, menthone, pulegone and carvone, are especially abundant^8^. Some components of this group of monoterpenes have been reported to possess anti-inflammatory activity^9^ that may justify, at least in part, the beneficial effects attributed to mint species by traditional and conventional medicine^10,11^. However, mint species exhibit many different chemotypes with significant diversity in qualitative and quantitative chemical composition^11,12^ that causes substantial variability, although poorly characterized, in terms of pharmacological activity of distinct plants and their essential oils. Besides differences related to distinct chemotypes, disparities in the experimental design, namely concerning the range of concentrations tested and the cell and animal models and inflammatory stimuli used, also make comparisons or prediction of the efficacy and potency of different plants, their essential oils and individual compounds impossible. This heterogeneity also makes it impossible to identify the structural determinants of activity, that is the structure-activity relationship (SAR) of this class of natural compounds.

The chemical optimization of an active compound requires that knowledge and is essential to improve its physicochemical properties and/or increase its potency and safety, thus yielding a suitable lead. This is especially important for monoterpenes whose volatility is a major drawback significantly limiting their use as active ingredients for the large scale production of medicines. Hence, elucidating the SAR is essential to guide the chemical modification of these compounds, namely to lower their vapour pressure at room temperature, without compromising pharmacological activity and/or increasing toxicity, and therefore to enable their progression towards new therapeutic agents^13^. Further, such knowledge is also essential to explain the different anti-inflammatory properties and potency of distinct mint chemotypes and their essential oils and can be used to predict the therapeutic potential of a given product based on its chemical composition.

**Figure 1:**
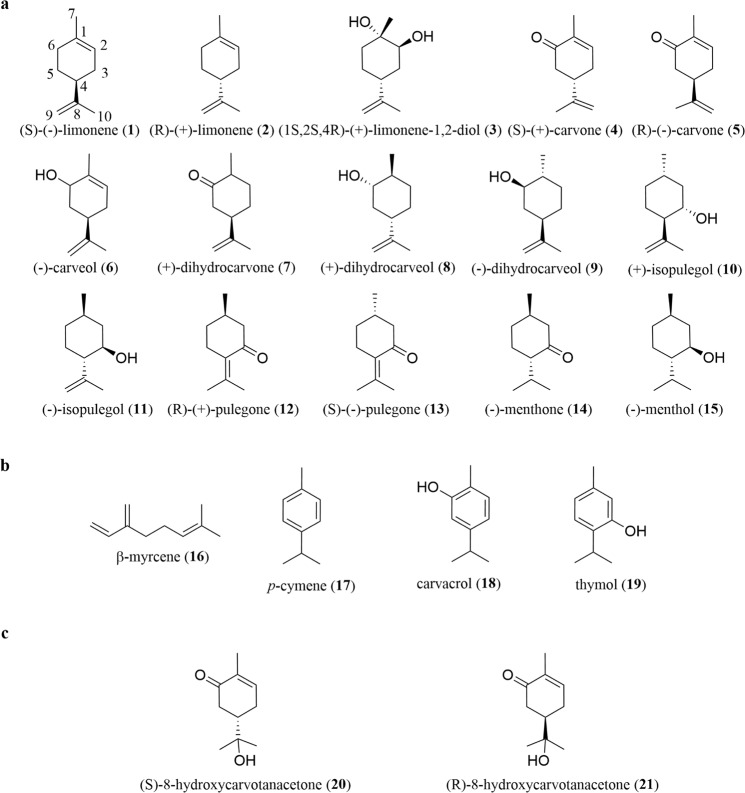
Structures of the monoterpenes tested. (**a**) Selected commercially available limonene-derived monoterpenes found in *Mentha* spp. (**b**) non-limonene-derived monoterpenes and (**c**) semi-synthetic limonene-derived monoterpenes were used to elucidate the role of specific chemical features. Stereochemistry of each chiral centre is indicated only where enantiomerically pure compounds were used. The numbering system employed here is based on compound **1**.

Thus, the purpose of this study was to assess, under standardized conditions, the anti-inflammatory activity of a selected group of monoterpenes belonging to the limonene synthase pathway that are abundant in mint species (Fig. 1a) and to compare the potency of the active ones by determining their half-maximal inhibitory concentrations (IC_50_). These data were then correlated with structural features to identify chemical determinants of activity and potency useful to enable chemical optimization of the active compounds.

For this, the ability of the test compounds to inhibit the production of nitric oxide (NO), a potent and destructive inflammatory mediator^14–16^, induced by bacterial lipopolysaccharide (LPS) in the mouse macrophage cell line, Raw 264.7, was used as a well-established primary screening assay for the identification of small molecules with anti-inflammatory activity^17,18^. Then and to further confirm their anti-inflammatory activity, we determined the ability of the two most potent compounds to inhibit the expression of NO synthase 2 (NOS2), the enzyme that produces large amounts of NO in response to inflammatory stimuli^15,19^, and interleukin-1β (IL-1β), two critical inflammatory mediators strongly associated with several acute and chronic human inflammatory diseases^3,16,20^.

Finally, the most potent compound identified in macrophages, S-(+)-carvone (**4**), was tested in primary human chondrocyte cultures treated with the pro-inflammatory and catabolic cytokine, IL-1β, as a widely used cell model of osteoarthritis (OA)^21^. This is the most common musculoskeletal disease, causing pain and loss of mobility and quality of life to millions of people worldwide^22^. While no curative therapies are yet available^23,24^ essential oils from *Mentha spicata*, which is especially abundant in S-(+)-carvone (**4**), are broadly used to decrease osteoarthritic pain^25^. Therefore, we hypothesized that such analgesic effect can be, at least in part, secondary to the anti-inflammatory properties of that compound. To test this hypothesis and evaluate its potential as an anti-osteoarthritic drug, we determined whether S-(+)-carvone (**4**) is also effective in reducing inflammatory responses in human chondrocytes.

## Results

### Nine out of twenty-one test compounds inhibit LPS-induced NO production in Raw 264.7 macrophages

Commercially available compounds with substituents in specific positions of the *p*-menthane skeleton in limonene were selected for the primary screening assay (Fig. 1a). We also tested four unrelated natural compounds (Fig. 1b), as well as two semi-synthetic carvone derivatives (Fig. 1c), to further elucidate the relevance to anti-inflammatory activity of specific chemical features of the limonene-derived compounds tested.

Various concentrations of the test compounds, in the absence or presence of LPS, were first evaluated for cytotoxicity using the resazurin reduction assay^26^ (Figs. S1 and S2). Concentrations above 400 µg/mL (approximately 2600 µM on average) were found not to be completely miscible in aqueous solution, even in the presence of 0.1% DMSO, and so this was the maximal concentration tested. Cytotoxicity was defined, according to the standard for cytotoxicity assessment, ISO 10993-5^27^, as the highest concentration that did not decrease cell viability by more than 30% relative to cells treated with LPS alone. Non-cytotoxic concentrations of each compound were then selected for the screening assay and subsequent studies.

To confirm the quality of the screening assay, we used Bay 11–7082, a selective IκB Kinase inhibitor that abrogates NF-κB activation^28^ and the expression of its target genes, including NOS2^29^, as a pharmacological control. Pre-treatment with 5 μM Bay 11-7082 decreased NO production to 48.1 ± 5.2% relative to cells treated with LPS alone, as expected.

At non-cytotoxic concentrations, none of the compounds tested affected basal NO production when added to macrophage cultures in the absence of LPS (data not shown) and eight (**1**, **2**, **10**, **11**, **12**, **14**, **15** and **16**) also had no effect on LPS-induced NO production (Table 1). Thirteen compounds were found to elicit a statistically significant decrease of LPS-induced NO production at the highest concentration tested, but of these, four (**3**, **17, 18** and **19**) had only a negligible effect of less than 20%. The other nine compounds (**4**, **5**, **6**, **7**, **8**, **9**, **13, 20** and **21**) elicited a robust inhibition greater than 30% (Table 1) and, therefore, were selected for further studies.

**Table 1:** Results of the primary screening assay relating the highest non-cytotoxic concentration tested and the % inhibition of LPS-induced NO production.

Compound	Highest non-cytotoxic concentration tested [µM (µg/mL)]	% inhibition of LPS-induced NO production^a^ [% ± SEM]	Adjusted *p* value^b^
(S)-(−)-limonene (**1**)	36.7 (5)	11.08 ± 4.65	0.1968
(R)-(+)-limonene (**2)**	367 (50)	−1.80 ± 0.55	0.4140
(1 S,2 S,4 R)-(+)-limonene-1,2-diol (**3**)	2349 (400)	13.90 ± 2.03	<0.0001
(S)-(+)-carvone (**4**)^c^	666 (100)	68.73 ± 1.55	<0.0001
(R)-(−)-carvone (**5**)^c^	666 (100)	71.50 ± 1.74	<0.0001
(−)-carveol (**6**)	2628 (400)	52.30 ± 5.54	0.0020
(+)-dihydrocarvone (**7**)	2628 (400)	73.57 ± 2.81	<0.0001
(+)-dihydrocarveol (**8**)	1297 (200)	71.03 ± 2.28	0.0002
(−)-dihydrocarveol (**9**)	2593 (400)	62.03 ± 8.62	0.0141
(+)-isopulegol (**10**)	2593 (400)	−18.47 ± 5.65	0.0002
(−)-isopulegol (**11**)	2593 (400)	−12.63 ± 1.30	<0.0001
(R)-(+)-pulegone (**12**)	1314 (200)	7.80 ± 2.98	0.1881
(S)-(−)-pulegone (**13**)	2628 (400)	35.60 ± 4.86	<0.0001
(−)-menthone (**14**)	1297 (200)	−9.88 ± 2.85	0.0041
(−)-menthol (**15**)	2560 (400)	−6.90 ± 1.24	0.0071
β-myrcene (**16**)	183 (25)	16.48 ± 17.00	0.1496
*p*-cymene (**17**)	37 (5)	8.50 ± 5.53	0.0100
carvacrol (**18**)	33 (5)	10.78 ± 1.34	<0.0001
thymol (**19**)	83 (12.5)	8.93 ± 1.91	0.0002
(S)-8-hydroxycarvotanacetone (**20**)	2378 (400)	78.43 ± 1.60	<0.0001
(R)-8-hydroxycarvotanacetone (**21**)	2378 (400)	76.25 ± 1.97	<0.0001

^**a**^NO production was assessed as the amount of nitrite (NO_2_^−^) accumulated in culture supernatants. % inhibition of NO production was calculated with the formula:

$$100-\left(\frac{(\,{[N{O}_{2}^{-}]}_{LPS}-{[N{O}_{2}^{-}]}_{test})\times 100 \% }{{[N{O}_{2}^{-}]}_{LPS}}\right)$$

where [NO_2_^−^]_LPS_ is the concentration of NO_2_^−^ in LPS-treated cells and [NO_2_^−^]_test_ is the concentration of NO_2_^−^ in cells treated with LPS in the presence of each test compound.

^**b**^Adjusted *p* value relative to LPS-treated cells.

^**c**^The highest non-cytotoxic concentration tested in the presence of LPS was 1331 µM (200 µg/mL). As 666 µM (100 µg/mL) decreased LPS-induced NO production to control levels, no further concentrations were tested in this primary screening assay.

Thus and to compare the active compounds in terms of potency, the respective concentration required to inhibit NO production by 50% (IC_50_) was determined by testing further non-cytotoxic concentrations. Since the maximal inhibition achieved with the highest non-cytotoxic concentration of (S)-(−)-pulegone (**13**) tested, did not exceed 36%, the IC_50_ for this compound was not determined. Results in Table 2 show that the order of potency of the remaining 8 active compounds is (S)-(+)-carvone (**4**)> (R)-(−)-carvone (**5**) ≫ (+)-dihydrocarveol (**8**)> (S)-8-hydroxycarvotanacetone (**20**)> (R)-8-hydroxycarvotanacetone (**21**)> (+)-dihydrocarvone (**7**)> (−)-carveol (**6**)> (−)-dihydrocarveol (**9**).

**Table 2:** IC_50_ values for the eight compounds found to significantly decrease LPS-induced NO production in the Raw 264.7 cell line.

Compound	IC_50_ [µM (µg/mL)]	95% CI^a^ [µM (µg/mL)]
(S)-(+)-carvone (**4**)	109.7 (16.50)	100.6–119.6 (15.13–17.98)
(R)-(−)-carvone (**5**)	122.8 (18.46)	113.8–132.7 (17.08–19.95)
(−)-carveol (**6**)	1997 (304.0)	1731–2303 (263.5–350.6)
(+)-dihydrocarvone (**7**)	1472 (224.0)	1105–1961 (168.2–298.4)
(+)-dihydrocarveol (**8**)	532.2 (82.13)	444.2–637.7 (68.54–98.41)
(−)-dihydrocarveol (**9**)	2141 (330.2)	1851–2475 (285.5–381.8)
(S)-8-hydroxycarvonatacetone (**20**)	762.0 (116.3)	670.4–866.0 (101.3–133.5)
(R)-8-hydroxycarvonatacetone (**21**)	841.5 (141.6)	776.0–912.4 (130.5–153.5)

^a^Concentration range within the 95% Confidence Interval (CI) of the IC_50_ value.

### Identification of chemical features relevant for activity by correlation with potency

Having determined the order of potency of the active compounds, we then correlated those results with structural features of all compounds tested to identify the relevant structural determinants of anti-inflammatory activity. For this, we defined a carbon numbering system applicable to all compounds tested, since their different functional groups and application of IUPAC rules would lead to different numbering of the same carbon atoms. Thus, IUPAC rules were used to define carbon numbering for limonene (Fig. 1) and the resulting numbering sequence was applied to all test compounds without considering their specific substituents. Besides limonene-derived compounds, four other natural monoterpenes, β-myrcene (**16**), *p*-cymene (**17**), carvacrol (**18**) and thymol (**19**) (Fig. 1b), were tested mainly to assess the relevance of the rigidity or flexibility of the molecule for anti-inflammatory activity. Additionally, two carvone derivatives (**20** and **21**, Fig. 1c) were synthesized and tested to assess the relevance of the isopropenyl group at C4.

A functional oxygenated group, either a carbonyl or a hydroxyl group, at C6 is present in all active compounds (**4-9**, **20** and **21**) and absent (**1** and **2)** or present at other positions (**3**, **10**–**12**, **14** and **15**) in all inactive or only slightly active (below 20% inhibition at the maximal non-cytotoxic concentration tested) compounds with the exception of (S)-(−)-pulegone (**13**) which bears a carbonyl group at C3 and showed weak activity.

Another important feature for activity seems to be the presence of an α,β double bond at C1, since its absence is the only difference between (+)-dihydrocarvone (**7**) and the much more potent carvone enantiomers (**4** and **5**). Moreover, the conjugation of this double bond to the carbonyl group at C6 also seems relevant for activity since the two most potent compounds, (S)-(+)-carvone (**4**) and (R)-(−)-carvone (**5**), present this feature which is also present in their derivatives (**20** and **21**), but not in the other less potent compounds. Nonetheless, (R)-(+)-pulegone (**12**) and (S)-(−)-pulegone (**13**) which have no or little activity, also have an α, β double bond conjugated to a carbonyl group, but involving the carbonyl group at C3 and the double bond at C4. Thus, the localization of the conjugated double bond and carbonyl group seems especially relevant for activity. Nevertheless, while (R)-(+)-pulegone (**12**) is inactive, its S enantiomer (**13**) showed weak activity, indicating that the stereochemistry can be relevant for activity.

Then and to elucidate the relevance of the isopropenyl group at C4, present in six active compounds (**4**, **5**, **6**, **7**, **8** and **9**), but also in five inactive ones (**1**, **2**, **3**, **10** and **11**), we synthesized derivatives of the two most potent compounds, the carvone enantiomers, where that group was replaced by a 2-hydroxyisopropanyl group. The 8-hydroxycarvotanacetone enantiomers (**20** and **21**) synthesized showed significant activity, although much lower than the respective parent compounds (**4** and **5**) (Table 2), thus confirming the relevance of the isopropenyl group at C4 for anti-inflammatory activity. Nonetheless, the isopropenyl group per se is not sufficient for activity, as compounds with such group, but lacking the oxygenated group at C6 (**1**, **2**, **3**, **10** and **11**) are inactive.

The results also show that (S)-(+)-carvone (**4**) and its derivative (**20**) are slightly more potent than their respective R isomers (**5** and **21**), from which they differ only in terms of stereochemistry at the chiral C4 atom. The third most potent compound used, (+)-dihydrocarveol (**8**), is a mixture of isomers of which the most abundant, (1 S,2 S,5 S)-dihydrocarveol, presents the S configuration at all its chiral centres, including C5 that corresponds to C4 of limonene, while its isomer, (−)-dihydrocarveol (**9**), four times less potent, is also a mixture of isomers, the most abundant of which, (1 R,2 R,5 R)-dihydrocarveol, presents the R configuration (detailed composition and purity of each test compound in Supplementary Table S1). Moreover, (−)-carveol (**6**) and (+)-dihydrocarvone (**7**) also have additional chiral centres at C6 or C1 and the products used are mixtures of S and R isomers at those positions, but in both, the most abundant is the isomer presenting the R configuration at the carbon atom corresponding to C4 (detailed composition in Supplementary Table S1). Similarly, (R)-(+)-pulegone (**12**) is inactive, while its S enantiomer (**13**) shows weak activity. Taken together, these results suggest that the S configuration, especially at C4, is more favourable for activity.

Finally, we tested four monoterpenes (**16**–**19**) unrelated to the limonene synthase pathway (Fig. 1b), but presenting various degrees of rigidity to elucidate the relevance of this feature for activity. Neither the more rigid (**17**–**19**), nor the more flexible (**16**) of these four compounds showed any activity.

Table 3 summarizes the chemical features found relevant for anti-inflammatory activity.

**Table 3:** Relationship between chemical features and potency of the nine active compounds.

Chemical features				Order of potency					
	4	>5	>>8*	>20	>21	>7*	>6*	>9*	>>13
=CO (C6)	+	+	−	+	+	+	−	−	−
–OH (C6)	−	−	+	−	−	−	+	+	−
=bond at C1	+	+	−	+	+	−	+	−	−
Michael centre	+	+	−	+	+	−	−	−	+
Michael centre sterically hindered	No	No	−	No	No	−	−	−	Yes
Isopropenyl group at C4	Yes	Yes	Yes	No	No	Yes	Yes	Yes	No
Chirality at C4	S	R	S	S	R	R	R	R	−
Chirality at C1	−	−	S	−	−	S/R	−	R	S
Chirality at C6	−	−	S	−	−	−	S/R	R	−

+/− denotes presence/absence; S/R denotes mixture of diastomers; *denotes the stereochemistry of the most abundant isomer in the compound tested. Detailed composition is provided in Supplementary Table S1.

### The carvone enantiomers inhibit LPS-induced NOS2 and IL-1β expression in macrophages

**Figure 2:**
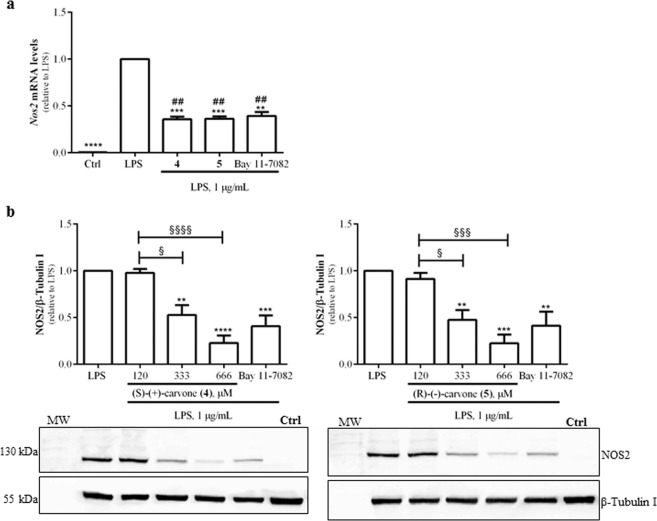
(S)-(+)-carvone (**4**) and (R)-(−)-carvone (**5**) decrease LPS-induced *Nos2* mRNA (**a**) and protein (**b**) levels in the Raw 264.7 cell line. Macrophage cultures were treated with 1 µg/mL LPS, for 6 h (**a**) or 18 h (**b**), following pre-treatment for 1 h with 666 µM of each test compound (**a**) or with the concentrations indicated in (**b**). As a positive control, the cells were similarly treated with the selective NF-κB inhibitor, Bay 11-7082, 5 μM. Control cells (Ctrl) were treated with the vehicle (0.1% DMSO) in the absence of LPS. Each column represents the mean ± SEM of four independent experiments. The blots shown are representative of, at least, three independent experiments and are cropped for clarity and conciseness. The corresponding full-length blots are presented in Fig. S3. **p < 0.01, ***p < 0.001 and ****p < 0.0001 relative to LPS-treated cells. ^##^*p* < 0.01 relative to the Ctrl. ^§^*p* < 0.05, ^§§§^*p* < 0.001 and ^§§§§^*p* < 0.0001 between the conditions indicated. MW: molecular weight marker.

**Figure 3:**
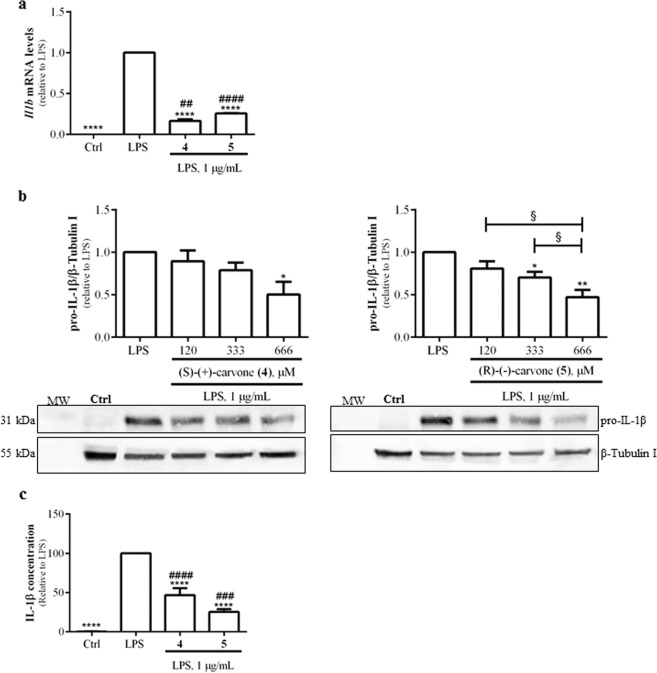
(S)-(+)-carvone (**4**) and (R)-(−)-carvone (**5**) decrease LPS-induced IL-1β mRNA (**a**) and protein (**b**,**c**) levels in the Raw 264.7 cell line. Macrophage cultures were treated with 1 µg/mL LPS, for 6 h (**a**) or 18 h (**b**,**c**), following pre-treatment for 1 h with 666 µM of each test compound (**a** and **c**) or with the concentrations indicated in (**b**). Control cells (Ctrl) were treated with vehicle (0.1% DMSO) in the absence of LPS. Each column represents the mean ± SEM of, at least, three independent experiments. The blots shown are representative of, at least, three independent experiments and are cropped for clarity and conciseness. The corresponding full-length blots are presented in Fig. S3. *p < 0.05, **p < 0.01 and ****p < 0.0001 relative to LPS-treated cells. ^##^*p* < 0.01, ^###^*p* < 0.001 and ^####^*p* < 0.0001 relative to the Ctrl. ^§^*p* < 0.05 between the conditions indicated. MW: molecular weight marker.

To further confirm the anti-inflammatory properties of the two most potent compounds, the expression of NOS2 and IL-1β were evaluated at the mRNA and protein levels. Macrophage treatment with 1 µg/mL LPS significantly increased NOS2 mRNA (Fig. 2a) and protein (Fig. 2b) levels which, as expected, were decreased by Bay 11-7082. The carvone enantiomers, (**4**) or (**5**), also significantly reduced LPS-induced NOS2 mRNA (Fig. 2a) and protein levels (Fig. 2b), as well as IL-1β mRNA levels (Fig. 3a). Upon transcription, this mRNA is translated into a precursor protein (pro-IL-1β) that undergoes partial hydrolysis by a proteolytic complex, the inflammasome, being converted into the mature IL-1β protein which is then secreted^30^. In agreement with the decrease in IL-1β mRNA levels, (S)-(+)-carvone (**4**) and (R)-(−)-carvone (**5**) significantly decreased the levels of both pro-IL-1β (Fig. 3b) and mature IL-1β secreted into the cell culture medium (Fig. 3c), relative to treatment with LPS alone.

**Figure 4:**
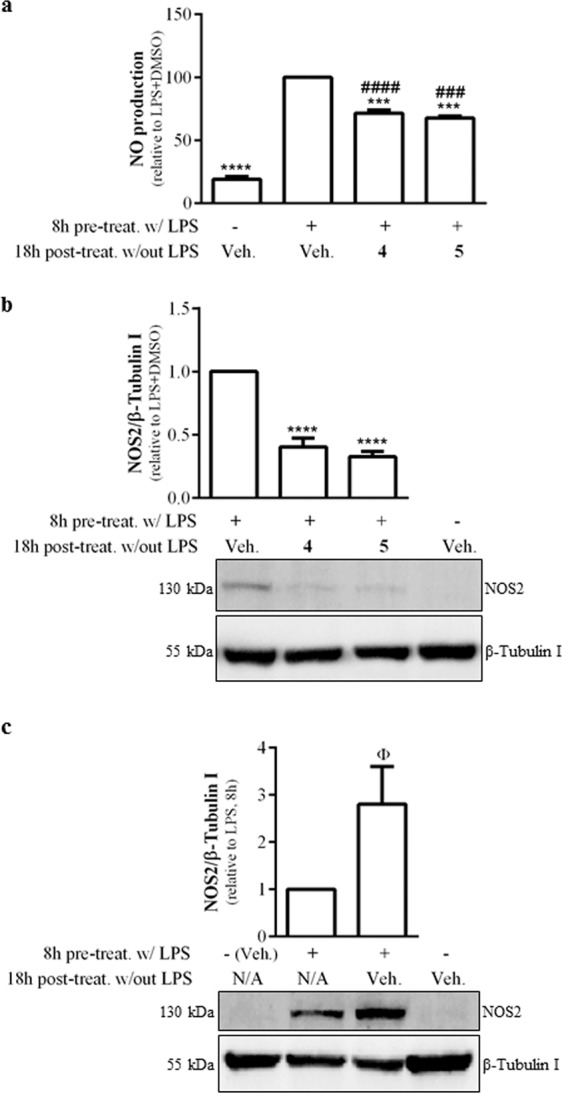
Effects of (S)-(+)-carvone (**4**) and (R)-(−)-carvone (**5**) on NO production and NOS2 protein levels pre-induced by treatment with LPS. In panels (a,b), macrophage cultures were treated with 1 μg/mL LPS, for 8 h to induce NOS2 expression. Then, the medium was changed to remove LPS and the cells were treated for another 18 h with 666 µM of each test compound or the vehicle (0.1% DMSO). Controls were set up by leaving the cells untreated for 8 h followed by addition of vehicle for 18 h. In panel c, cells were pre-treated with LPS or left untreated for 8 h and immediately processed for protein extraction or treated with or without LPS for 8 h and then further incubated with vehicle for another 18 h. Each column represents the mean ± SEM of, at least, three independent experiments. The blots shown are representative of, at least, three independent experiments and are cropped for clarity and conciseness. The corresponding full-length blots are presented in Fig. S3. ***p < 0.001 and ****p < 0.0001 relative to LPS, 8 h + vehicle, 18 h. ^###^*p* < 0.001 and ^####^*p* < 0.0001 relative to untreated cells, 8 h + DMSO, 18 h. ^Φ^*p* < 0.05 relative to LPS, 8 h. N/A: not applicable.

To further characterize the mechanism of action of the carvone enantiomers (**4** and **5**), we determined whether they are also effective when added to the cells after the inflammatory stimulus and the mechanism involved, that is, whether they act by modifying NOS2 protein levels and/or its enzyme activity. For this, we treated macrophages with LPS for 8 h to induce NOS2 expression and protein synthesis. Then, the cells were washed to remove LPS and new medium with the carvone enantiomers (**4** and **5**) was added to the respective wells for 18 h. The results in Fig. 4a show that treatment with either compound decreased NO production by approximately 30% while NOS2 protein levels were reduced by approximately 60% (Fig. 4b). This suggests that the decrease in NO production is secondary to the decrease in NOS2 protein and not to a direct inhibition of the enzyme activity.

To determine whether this decrease was due to inhibition of NOS2 protein synthesis, still occurring after removal of LPS, or to induction of its degradation, we evaluated its protein levels 8 h after treatment with LPS. The results obtained show that NOS2 protein levels still increased after removal of LPS (Fig. 4c), indicating that NOS2 protein synthesis continues even after removal of the inducing stimulus. This indicates that the decrease in NO production observed in Fig. 4a is not due to inhibition of NOS2 enzyme activity, but rather to inhibition of its synthesis.

### (S)-(+)-carvone (4) inhibits inflammatory responses induced by IL-1β in human chondrocytes

**Figure 5:**
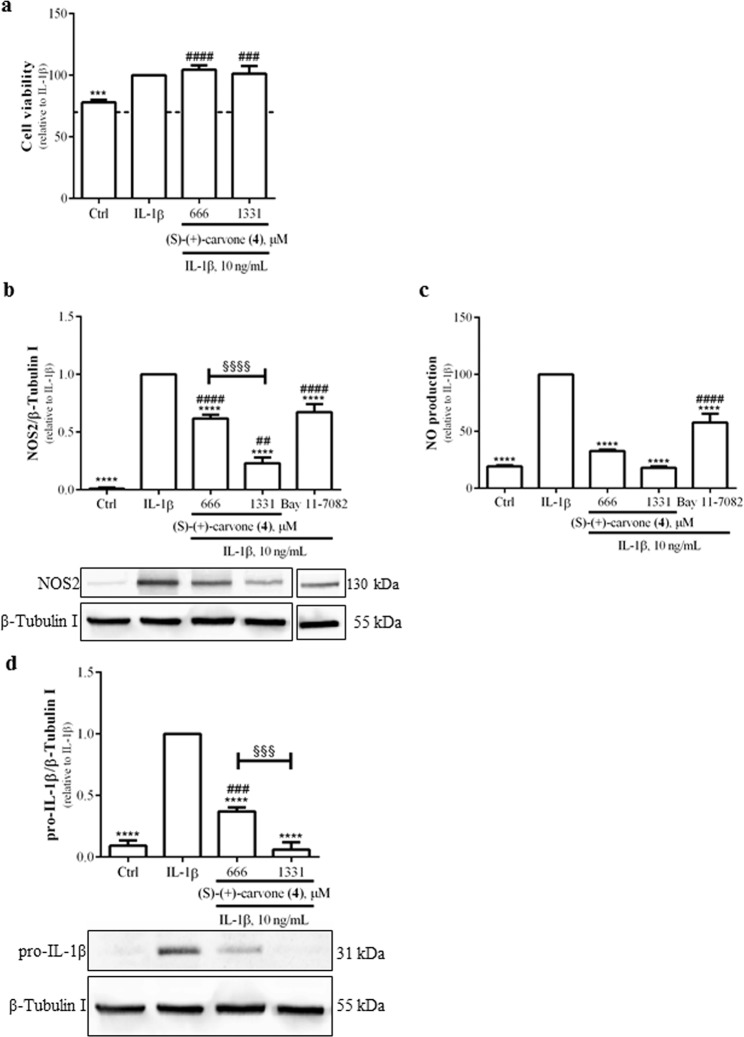
(S)-(+)-carvone (**4**) does not affect cell viability (**a**) and decreases IL-1β-induced NOS2 protein levels (**b**) and NO production (**c**) as well as pro-IL-1β protein levels (**d**) in human chondrocytes. The cells were treated with 10 ng/mL IL-1β for 24 h (**a**, **b**, **c** and **d**), following pre-treatment for 1 h with 666 or 1331 µM of (S)-(+)-carvone (**4**). As a positive control, the cells were similarly treated with 5 μM Bay 11-7082 (**b** and **c**). Control cells (Ctrl) were treated with the vehicle (0.1% DMSO) in the absence of IL-1β. Each column represents the mean ± SEM of, at least, three independent experiments. The blots shown are representative of, at least, three independent experiments and are cropped for clarity and conciseness. The blots shown in Fig. 5b were vertically sliced before the last condition (Bay 11-7082) to exclude a condition not relevant for the present study. The corresponding full-length blots are presented in Fig. S3. ***p < 0.001 and ****p < 0.0001 relative to IL-1β-treated cells. ^##^*p* < 0.01, ^###^*p* < 0.001 and ^####^*p* < 0.0001 relative to the Ctrl. ^§§§^*p* < 0.001 and ^§§§§^*p* < 0.0001 between the conditions indicated.

First, the highest concentrations of (S)-(+)-carvone (**4**) not toxic to murine macrophages were tested in human chondrocytes and found not to affect cell viability relative to cells treated with IL-1β alone (Fig. 5a). At the same concentrations, (S)-(+)-carvone (**4**) significantly decreased IL-1β-induced NOS2 protein levels (Fig. 5b) and NO production (Fig. 5c) in human chondrocytes. 5 µM Bay 11-7082 significantly decreased both parameters (Fig. 5b,c), confirming the quality of the model system. Finally, (S)-(+)-carvone (**4**) also significantly decreased pro-IL-1β protein levels in a concentration-dependent manner (Fig. 5d).

## Discussion

To the best of our knowledge and among the nine active compounds, (S)-(+)-carvone (**4**), (+)-dihydrocarvone (**7**), (+)- (**8**) and (−)-dihydrocarveol (**9**), (S)-(−)-pulegone (**13**) and the carvone derivatives (**20** and **21**), were never reported to have anti-inflammatory effects, while carvone, either as the racemic mixture^31^ or the (R)-(−) enantiomer (**5**)^3,32,33^ and (−)-carveol (**6**)^33^, were recently reported to inhibit some effects correlated with inflammation.

Among the compounds that showed no activity, the lack of inhibition of LPS-induced NO production by the limonene enantiomers (**1** and **2**), (R)-(+)-pulegone (**12**), (−)-menthone (**14**), (−)-menthol (**15**), β-myrcene (**16**), *p*-cymene (**17**), carvacrol (**18**) and thymol (**19**) contrasts with other reports that suggest anti-inflammatory activity for these compounds^34–42^. At least in part, these discrepancies can be due to the use of distinct models, namely cell lines, animal models and endpoints analysed, as well as to different concentrations tested. Significant anti-inflammatory effects were recently reported for limonene (racemic mixture, up to 200 μg/mL) and β-myrcene (up to 50 μg/mL) in human chondrocytes^37^, as well as in the Raw 264.7 cell line^41,42^, but at concentrations much higher than those used in the current study. These discrepancies can be due in part to the use of distinct methods to assess cell viability, namely the lactate dehydrogenase (LDH) release assay, based on the integrity of the plasma membrane^43^, used in the studies by Kim *et al*. (2013) and Yoon *et al*. (2010), versus the resazurin or the MTT reduction assays dependent on the integrity and activity of mitochondria^44^, used in our studies. Nonetheless, since cytotoxicity to human chondrocytes and murine macrophages was evaluated by similar methods, the much lower cytotoxic concentrations observed in mouse macrophages indicate that these cells are more sensitive to cytotoxicity induced by those monoterpenes, suggesting that their effects are species and/or cell type-specific, which further highlights the importance of standardized side-by-side comparisons of different compounds.

Having performed such a standardized comparison, some chemical features were identified as relevant for activity and potency (Table 3). Among those, the presence of a functional oxygenated group at C6 appears as the major determinant for activity while the conjugation of the carbonyl group at that position with the α,β double bond at C1, present in the carvone enantiomers (**4** and **5**) and their derivatives (**20** and **21**), was found to further increase potency. Such a conjugation provides a Michael acceptor site, that is, an electrophilic site due to the presence of an electron-withdrawing group in close proximity to a double bond, which is a chemical feature relevant for interaction with biomolecules, namely proteins^45,46^, and thus likely relevant for interaction of these molecules with their target. However, the pulegone enantiomers (**12** and **13**) which have no or little activity, also have an α,β double bond conjugated to a carbonyl group, but involving the carbonyl group at C3 and the double bond of the isopropylidene group at C4. In this case, the proximity of the methyl group at C10 to the carbonyl group at C3 can cause a sterical hindrance impairing the interaction with the molecular target^46^ and thus impeding activity.

The lower potency of the carvone derivatives (**20** and **21**) relative to the parent compounds (**4** and **5**) can only be due to the replacement of the isopropenyl at C4 by a 2-hydroxyisopropanyl group. The presence of the hydroxyl group can provide an additional hydrogen binding site, thus creating a new site for chemical interactions that may negatively impact anti-inflammatory activity. Furthermore, this hydroxyl group also increases the volume of the molecule at that region which can hinder access to the pharmacological target and thus decrease the activity of those compounds. Replacement of the isopropenyl group at C4 can also explain why these two compounds are less potent than the third most potent compound, (+)-dihydrocarveol (**8**), which despite bearing a hydroxyl group at C6, retains the isopropenyl group at C4.

Another relevant feature for activity must be chirality as, otherwise, all the enantiomer pairs tested should have similar activities which is not the case. Moreover, 3 of the active compounds (**6**-**8**) have additional chiral centres at C1 and/or C6. Interestingly, the order of potency of the nine active compounds is closely related to the S configuration of the chiral atom at C4, although other chemical features, namely the presence of a functional oxygenated group at C6, the double bond at C1 and the isopropenyl group at C4, seem more relevant (Table 3). Since the S or R configurations significantly affect the 3D conformation of a molecule, the S conformation at C4 by conferring some planarity to that region of the molecules is likely relevant for access to and interaction with the target.

Finally, we tested four monoterpenes (Fig. 1b) unrelated to the limonene synthase pathway, but presenting various degrees of rigidity to elucidate the relevance of this feature for activity. None of these four compounds showed activity, including carvacrol (**18**) even though it bears a hydroxyl group at C6. Unlike the nine active compounds, including those bearing a hydroxyl group at the same position (**6**, **8** and **9**), the cyclohexane ring in carvacrol (**18**) is aromatic suggesting that its rigidity impairs the interaction with the target, leading to almost no activity. Likewise, thymol (**19**) which differs from carvacrol only in the position of the hydroxyl group, and *p*-cymene (**17**) which has no functional groups, also showed no activity. On the other hand, β-myrcene (**16**), an aliphatic compound representing a flexible structure, also showed no activity, suggesting that too flexible (**16**) or too rigid (**17, 18** and **19**) structures are unfavourable for activity.

In summary, the results obtained indicate that higher potency is conferred by 1) the carbonyl group at C6, rather than the hydroxyl group, 2) the presence of a Michael centre resulting from the conjugation of an α,β double bond at C1 to a carbonyl group at C6, 3) an isopropenyl group at C4 or, at least, the absence of hydrogen binding sites and bulky groups at that position, and 4) the S configuration, especially at C4. These findings can be useful to predict the anti-inflammatory activity of distinct mint species and their chemotypes once their composition in limonene-derived monoterpenes is known.

As found for inhibition of NO production, the two most potent compounds found, the carvone enantiomers, also significantly decreased the mRNA and protein levels of NOS2 (Figs. 2 and 4) and IL-1β (Fig. 3), further strengthening their anti-inflammatory activity and suggesting that they act at the transcriptional or pre-transcriptional levels. Moreover, (S)-(+)-carvone (**4**) was found to have similar anti-inflammatory effects in human chondrocytes. This indicates that (S)-(+)-carvone (**4**) is not only effective in inhibiting LPS-induced inflammatory responses in macrophages, but also efficiently inhibits the responses induced by a distinct inflammatory and catabolic stimulus in a different cell type. The anti-inflammatory effects of (S)-(+)-carvone (**4**) in human chondrocytes are especially relevant because inflammatory cytokines, like IL-1β, drive joint destruction by inducing the expression of catabolic enzymes^47^ and also contribute to OA pain^48^, namely by inducing the expression of nerve growth factor by synovial macrophages^49^. Moreover, unlike other monoterpenes, e.g. limonene, (S)-(+)-carvone (**4**) is effective as an anti-inflammatory agent both in macrophages and human chondrocytes at similar concentrations. Given the relevance of both cell types and the inflammatory stimuli used to OA pathophysiology^23,47^, (S)-(+)-carvone (**4**) may be efficient in halting joint destruction in OA and also contribute to reduce pain. Future studies will aim at further elucidating its mechanism of action and evaluating its anti-osteoarthritic properties *in vivo*.

## Methods

### Test compounds

Test compounds **1**–**17** and **19** were from Sigma-Aldrich Co. (St Louis, MO, USA). Thymol (**18**) was from British Drug Houses. Compounds **20** and **21** were synthesized at our laboratory, as described below. Details about purity and isomer composition are provided in Supplementary Table S1.

### Chemical synthesis of compounds 20 and 21

The synthetic procedure was adapted from Buechi and Wueest (1979).

#### Synthesis of (S)-5-(2-hydroxypropan-2-yl)-2-methylcyclohex-2-en-1-one [(S)-8-hydroxycarvotanacetone, **20**]

1 mL of 50% aqueous sulphuric acid was slowly added to 150 mg (1 mmol) of (S)-(+)-carvone (**4**) at 0 °C. The mixture was stirred for 24 h at 0 °C. After extraction with 2 mL of hexane-ether (3:1), the aqueous layer was extracted with diethyl ether (6 × 2 mL) for 24 h. The ether solution was washed with brine containing sodium bicarbonate, dried over anhydrous sodium sulphate and evaporated under reduced pressure. The remaining aqueous layer was extracted with ethyl acetate (3 × 2 mL) for 12 h. The organic phases were washed with brine containing sodium bicarbonate, dried over anhydrous sodium sulphate and evaporated under reduced pressure. The combined crude extract was purified by flash chromatography (hexane: ethyl acetate 1:1) to give compound **20** (80 mg, 48% yield) as a viscous liquid. Purity (GC-MS): 99.7%. ^1^H NMR (400 MHz, CDCl_3_) δ: 6.77 (m, 1 H), 2.64-2.42 (m, 1 H), 2.28-2.21 (m, 1 H), 2.28-2.21 (m, 2 H), 1.78 (s, 3 H), 1.24 (s, 3 H), 1.23 (s, 3 H). ^13^C NMR (101 MHz, CDCl_3_) δ: 200.35 (C=O), 145.15 (CH), 135.24 (CH), 71.64 (C-OH), 46.05 (CH), 39.61 (CH_2_), 27.31 (CH_3_), 27.25(CH_2_), 27.02(CH_3_), 15.61(CH_3_). IR (ATR) cm^-1^: 3424, 2973, 2925, 2891, 1656, 1381, 1367, 1143, 1111, 1059, 928, 903,813, 711, 678. MS m/z: 28.1, 43.0, 59.1, 95.0, 110.1, 135.1, 150.1, 168.1.

#### Synthesis of (R)-5-(2-hydroxypropan-2-yl)-2-methylcyclohex-2-en-1-one [(R)-8-hydroxycarvotanacetone, **21**]

Compound **21** was synthesized as described for compound **20** using as starting material (R)-(−)-carvone (**5**). The combined crude was purified by flash chromatography (hexane: ethyl acetate 1:1) to give compound **21** (78 mg, 48% yield) as a viscous liquid. Purity (GC-MS): 99.5%. ^1^H NMR (400 MHz, CDCl_3_) δ: 6.77 (m, 1 H), 2.64-2.42 (m, 1 H), 2.28-2.21 (m, 1 H), 2.28-2.21 (m, 2 H), 1.78 (s, 3 H), 1.24 (s, 3 H), 1.23 (s, 3 H). ^13^C NMR (101 MHz, CDCl_3_) δ: 200.35 (C=O), 145.15 (CH), 135.24 (CH), 71.64 (C-OH), 46.05 (CH), 39.61 (CH_2_), 27.31 (CH_3_), 27.25(CH_2_), 27.02(CH_3_), 15.61(CH_3_). IR (ATR) cm^-1^: 3424, 2973, 2925, 2891, 1656, 1381, 1367, 1143, 1111, 1059, 928, 903,813, 711, 678. MS m/z: 28.1, 43.0, 59.1, 95.0, 110.1, 135.1, 150.1, 168.1.

### Cell culture and treatment

#### Macrophages

The mouse macrophage cell line, Raw 264.7 (ATCC No. TIB-71), was cultured in DMEM supplemented with 10% non-heat inactivated foetal bovine serum (FBS), 100 U/mL penicillin and 100 µg/mL streptomycin. Raw 264.7 cells were plated at a density of 3 × 10^5^ cells/mL and left to stabilize for up to 24 h.

#### Human chondrocytes

Human knee cartilage was collected within 24 h of death from the distal femoral condyles of multi-organ donors (48–77 years old, mean = 65, n = 7) at the Bone and Tissue Bank of the University and Hospital Centre of Coimbra (CHUC). Only waste tissue resulting from the preparation of bone tissue for cryopreservation was used. All procedures were approved by the Ethics Committee of CHUC (protocol approval number 8654/DC), which follows the Declaration of Helsinki and Oviedo Convention and the Portuguese legislation for organ donation.

Chondrocytes were isolated by enzymatic digestion from cartilage samples as previously described^3^. Briefly, cartilage shavings underwent sequential digestion with Pronase (Roche, Indianapolis, IN, USA) and collagenase A (Roche, Indianapolis, IN, USA). To avoid chondrocyte dedifferentiation, non-proliferating monolayer cultures were setup by plating 1× 10^6^ chondrocytes/mL in HAM: F12 medium containing 3% antibiotics and 5% FBS and allowed to recover for 24 h at 37 °C in a humidified atmosphere with 5% CO_2_. Prior to any treatments, chondrocytes were serum-starved overnight and thereafter maintained in culture medium without FBS.

#### Cell treatments

Test compounds and Bay 11-7082 (Calbiochem, San Diego, CA, USA), used as a pharmacological control, were dissolved in dimethyl sulfoxide (DMSO; Sigma-Aldrich Co) so that its final concentration in the culture medium did not exceed 0.1% (v/v). This vehicle was used as control. Lipopolysaccharides from Escherichia coli 026:B6 (LPS; Sigma-Aldrich Co.) were dissolved in phosphate buffered saline (PBS). Recombinant human interleukin-1β (IL-1β; Peprotech, Rocky Hill, NJ, USA) was dissolved in PBS containing 0.1% Bovine Serum Albumin. Test compounds or the vehicle were added to macrophage cell cultures or human chondrocytes 1 h before the pro-inflammatory stimulus, 1 µg/mL LPS or 10 ng/mL IL-1β respectively, and maintained for the rest of the experimental period, except for experiments in Fig. 4 (details in the Results section and figure legend). The concentrations of each compound and the experimental treatment periods are indicated in figure legends.

### Selection of non-cytotoxic concentrations by the resazurin reduction assay

Resazurin is a redox dye used as an indicator of cellular metabolic activity for various applications, namely cell viability, proliferation and toxicity. The assay is based on the intracellular reduction of the non-fluorescent resazurin to resorufin (a fluorescent and pink coloured compound) by mitochondrial or microsomal enzymes that use NADH or NADPH as electron sources. Since only metabolically active cells can reduce the dye, the increase in fluorescence or absorbance is directly proportional to the number of viable cells^26,50^.

To select non-cytotoxic concentrations of the test compounds, the resazurin solution was added to each well to a final concentration of 50 µM, 90 min before the end of the treatment period indicated in the figure legends. Then, absorbances at 570 nm and 620 nm (reference wavelength) were read in a Biotek Synergy HT plate reader (Biotek, Winooski, VT, USA).

### Nitric oxide production

NO production was measured as the amount of nitrite accumulated in the culture supernatants using the Griess reaction which is based in the reaction of nitrite with sulfanilamide under acidic conditions, yielding a diazonium ion that couples to N-(1-napthtyl)ethylenediamine dihydrochloride to form a water-soluble red-violet azo dye that absorbs at 550 nm^51^. Briefly, equal volumes of culture supernatants and reagents [equal volumes of 1% (w/v) sulphanilamide in 5% (v/v) phosphoric acid and 0.1% (w/v) N-(1-napthtyl)ethylenediamine dihydrochloride] were mixed and incubated for 10 min, at room temperature, in the dark. The concentration of nitrite accumulated in the culture supernatants was calculated by interpolation of the absorbance of each sample, read in Biotek Synergy HT plate reader (Biotek), in a standard curve of sodium nitrite.

### Total RNA extraction and quantitative real-time PCR (qRT-PCR)

Total RNA extraction and qRT-PCR were performed as described before^52^. Briefly, total RNA was extracted using the NZYol (NZYTECH, Lisbon, Portugal) and quantified in a NanoDrop ND-1000 spectrophotometer at 260 nm. RNA purity was assessed by analysis of 260/230 and 260/280 absorption ratios. The cDNA was reverse-transcribed using NZY First Strand cDNA Synthesis Kit (NZYTECH), beginning with 2 µg of total RNA. qRT-PCR was performed, in duplicate for each sample, using NZYSpeedy qPCR Green Master Mix (2×) (NZYTECH) on CFX96 Real-Time PCR Detection System (Bio-Rad, Hercules, CA, USA).

The efficiency of the amplification reaction for each gene was calculated using a standard curve of a series of diluted cDNA samples and the specificity of the amplification products was assessed by analysing the melting curve generated in the process.

Gene expression changes were analysed using the built-in CFX Manager software which enables the analysis of the results by the Pfaffl method, a variation of the ΔΔCT method corrected for gene-specific efficiencies^53^. The results were normalized using *Hprt1* as housekeeping gene. This gene was experimentally determined with Genex software using NormFinder and geNorm algorithms (MultiD Analyses AB, Göteberg, Sweden) as the most stable for the treatment conditions used. Specific sets of primers for *Nos2*, *Il1b* and *Hprt1* (Table 4) were designed using Beacon Designer software version 8 (Premier Biosoft International, Palo Alto, CA, USA).

**Table 4:** Oligonucleotide Primer Pairs Used for qRT-PCR.

Gene name	Genbank accession number	Forward sequence	Reverse sequence
*Hprt-1*	NM_013556	GTTGAAGATATAATTGACACTG	GGCATATCCAACAACAAAC
*Nos2*	NM_010927	GCTGTTAGAGACACTTCTGAG	CACTTTGGTAGGATTTGACTTTG
*Il1b*	NM_008361	ACCTGTCCTGTGTAATGAAAG	GCTTGTGCTCTGCTTGTG

### Western blotting

Total cell extracts were prepared and western blot was performed as described before^54^. Briefly, total (25 µg for Raw 264.7 cell line and 20 µg for human chondrocytes) proteins were separated by SDS-PAGE under reducing conditions and electrotransferred onto PVDF membranes. These were probed overnight at 4 °C or for 2 h at room temperature with rabbit polyclonal antibody against IL-1β (dilution 1:500; sc-7884, Santa Cruz Biotechnology, INC., Texas, USA) or mouse monoclonal antibody NOS2 (dilution 1:500; MAB9502, R&D Systems, Minneapolis, MN, USA) and then with anti-rabbit or anti-mouse alkaline phosphatase-conjugated secondary antibodies (dilution 1:20000; GE Healthcare, Chalfont St. Giles, UK) for 1 h at room temperature. Immune complexes were detected with Enhanced ChemiFluorescence reagent (GE Healthcare) in the imaging system Thyphoon FLA 9000 (GE Healthcare). The membranes were reprobed with a mouse monoclonal anti-β-Tubulin I antibody (Sigma-Aldrich Co.), diluted at 1:20000, as a loading control, for 1 h at room temperature. Image analysis was performed with TotalLab TL120 software (Nonlinear Dynamics Ltd).

### Measurement of secreted IL-1β

The concentration of IL-1β in the culture supernatants was measured using the Mouse IL-1β ELISA kit (ThermoScientific, Rockford, USA), following the manufacturer’s instructions.

### Statistical Analysis

Results are presented as means ± SEM. Statistical analysis using GraphPad Prism version 6.0 (GraphPad Software, San Diego, CA, USA). Normal distribution of the data was evaluated with the D’Agostino & Pearson omnibus, the Shapiro-Wilk and the Kolmogorov-Smirnov tests. In cases where the number of samples is too small, we assumed the data follow a normal distribution, as this was verified in all cases where the sample number was larger, including analysis of the same analyte under different experimental treatments. Statistical analysis was performed by one-way ANOVA with the Dunnett post-test for comparison to a control group and the Tukey post-test for multiple comparisons, except in Fig. 4c where the unpaired t-test was used to compare a specific condition with its respective control. Results were considered statistically significant at *p* < 0.05.

## Supplementary information

Supplementary information.

## Data Availability

The datasets generated and analysed during the current study are available from the corresponding author on reasonable request.

## References

[1] Antonelli M, Kushner I (2017). It’s time to redefine inflammation. FASEB J..

[2] Kotas ME, Medzhitov R (2015). Homeostasis, inflammation, and disease susceptibility. Cell.

[3] Rosa SC, Judas F, Lopes MC, Mendes AF (2008). Nitric oxide synthase isoforms and NF-kappaB activity in normal and osteoarthritic human chondrocytes: regulation by inducible nitric oxide. Nitric Oxide.

[4] Nathan C, Ding A (2010). Nonresolving inflammation. Cell.

[5] Killeen MJ, Linder M, Pontoniere P, Crea R (2014). NF-kappabeta signaling and chronic inflammatory diseases: exploring the potential of natural products to drive new therapeutic opportunities. Drug Discov. Today.

[6] Ghosh N (2016). Chronic Inflammatory Diseases: Progress and Prospect with Herbal Medicine. Curr. Pharm. Des..

[7] Dinarello CA (2010). Anti-inflammatory Agents: Present and Future. Cell.

[8] Mimica-Dukic N, Bozin B, Mentha L (2008). species (Lamiaceae) as promising sources of bioactive secondary metabolites. Curr. Pharm. Des.

[9] de Cassia da Silveira e Sa R, Andrade LN, de Sousa DP (2013). A review on anti-inflammatory activity of monoterpenes. Molecules.

[10] Karousou R, Balta M, Hanlidou E, Kokkini S (2007). “Mints”, smells and traditional uses in Thessaloniki (Greece) and other Mediterranean countries. J. Ethnopharmacol..

[11] Fatiha Brahmi, M. K., Chibane M. & Duez P. In *Aromatic and Medicinal Plants - Back to Nature* (ed. Hany A. El-Shemy) 47–80 (IntechOpen, 2017).

[12] Singh P, Pandey AK (2018). Prospective of Essential Oils of the Genus Mentha as Biopesticides: A Review. Front. Plant Sci..

[13] Hughes JP, Rees S, Kalindjian SB, Philpott KL (2011). Principles of early drug discovery. Br. J. Pharmacol..

[14] Chung HY (2011). Molecular inflammation as an underlying mechanism of the aging process and age-related diseases. J. Dent. Res..

[15] Nagy G, Clark JM, Buzas EI, Gorman CL, Cope AP (2007). Nitric oxide, chronic inflammation and autoimmunity. Immunology Lett..

[16] Thomas DD, Wink DA (2017). NOS2 as an Emergent Player in Progression of Cancer. Antioxid. Redox Signal..

[17] Yu Q (2016). Resokaempferol-mediated anti-inflammatory effects on activated macrophages via the inhibition of JAK2/STAT3, NF-kappaB and JNK/p38 MAPK signaling pathways. International Immunopharmacol.

[18] Kumar RP, Abraham A (2017). Inhibition of LPS induced pro-inflammatory responses in RAW 264.7 macrophage cells by PVP-coated naringenin nanoparticle via down regulation of NF-kappaB/P38MAPK mediated stress signaling. Pharmacol. Rep..

[19] Forstermann U, Kleinert H (1995). Nitric oxide synthase: expression and expressional control of the three isoforms. Naunyn Schmiedebergs Arch. Pharmacol..

[20] Asiimwe N, Yeo SG, Kim MS, Jung J, Jeong NY (2016). Nitric Oxide: Exploring the Contextual Link with Alzheimer’s Disease. Oxid. Med. Cell. Longev..

[21] Johnson CI, Argyle DJ, Clements DN (2016). *In vitro* models for the study of osteoarthritis. Vet. J..

[22] Cross M (2014). The global burden of hip and knee osteoarthritis: estimates from the global burden of disease 2010 study. Ann. Rheum. Dis..

[23] Goldring MB, Berenbaum F (2015). Emerging targets in osteoarthritis therapy. Curr. Opin. Pharmacol..

[24] Martel-Pelletier J (2016). Osteoarthritis. Nat. Rev. Dis. Primers.

[25] Mahboubi M (2017). Mentha spicata as natural analgesia for treatment of pain in osteoarthritis patients. Complement. Ther. Clin. Pract.

[26] O’Brien J, Wilson I, Orton T, Pognan F (2000). Investigation of the Alamar Blue (resazurin) fluorescent dye for the assessment of mammalian cell cytotoxicity. Eur. J. Biochem..

[27] Standardization, I. O. f. In *Part 5: Tests for in vitro cytotoxicity* Vol. ISO 10993-5:2009(E) 1–34 (IHS, Switzerland, 2009).

[28] Pierce JW (1997). Novel inhibitors of cytokine-induced IkappaBalpha phosphorylation and endothelial cell adhesion molecule expression show anti-inflammatory effects *in vivo*. J. Biol. Chem..

[29] Mendes Sdos S (2009). Microarray analyses of the effects of NF-kappaB or PI3K pathway inhibitors on the LPS-induced gene expression profile in RAW264.7 cells: synergistic effects of rapamycin on LPS-induced MMP9-overexpression. Cell. Signal..

[30] Patel MN (2017). Inflammasome Priming in Sterile Inflammatory Disease. Trends Mol. Med..

[31] Sepúlveda-Arias JC, Veloza LA, Escobar LM, Orozco LM, Lopera IA (2013). Anti-inflammatory effects of the main constituents and epoxides derived from essential oils obtained from Tagetes lucida, Cymbopogon citratus, Lipia alba and Eucalyptus citriodora. Journal of Essential Oil Research.

[32] Abe S (2003). Suppression of tumor necrosis factor-alpha-induced neutrophil adherence responses by essential oils. Mediators Inflamm..

[33] Marques FM (2019). *In vitro* anti-inflammatory activity of terpenes via suppression of superoxide and nitric oxide generation and the NF-kappaB signalling pathway. Inflammopharmacology.

[34] Ghasemi-Pirbaluti M, Motaghi E, Bozorgi H (2017). The effect of menthol on acute experimental colitis in rats. Eur. J. Pharmacol..

[35] Li Z, Hua C, Pan X, Fu X, Wu W (2016). Carvacrol Exerts Neuroprotective Effects Via Suppression of the Inflammatory Response in Middle Cerebral Artery Occlusion Rats. Inflammation.

[36] Nagoor Meeran MF, Jagadeesh GS, Selvaraj P (2015). Thymol attenuates inflammation in isoproterenol induced myocardial infarcted rats by inhibiting the release of lysosomal enzymes and downregulating the expressions of proinflammatory cytokines. Eur. J. Pharmacol..

[37] Rufino AT (2015). Evaluation of the anti-inflammatory, anti-catabolic and pro-anabolic effects of E-caryophyllene, myrcene and limonene in a cell model of osteoarthritis. Eur. J. Pharmacol..

[38] Xue J (2015). L-Menthone confers antidepressant-like effects in an unpredictable chronic mild stress mouse model via NLRP3 inflammasome-mediated inflammatory cytokines and central neurotransmitters. Pharmacol. Biochem. Behav..

[39] Yao QS, Chiou GC (1993). Inhibition of crystallins-induced inflammation in rabbit eyes with five phytogenic compounds. Zhongguo Yao Li Xue Bao.

[40] Choi YY, Kim MH, Lee H, Jo SY, Yang WM (2018). R)-(+)-pulegone suppresses allergic and inflammation responses on 2,4-dinitrochlorobenzene-induced atopic dermatitis in mice model. J. Dermatol. Sci..

[41] Yoon WJ, Lee NH, Hyun CG (2010). Limonene suppresses lipopolysaccharide-induced production of nitric oxide, prostaglandin E2, and pro-inflammatory cytokines in RAW 264.7 macrophages. J. Oleo Sci.

[42] Kim KN (2013). Anti-inflammatory effect of essential oil and its constituents from fingered citron (Citrus medica L. var. sarcodactylis) through blocking JNK, ERK and NF-kappaB signaling pathways in LPS-activated RAW 264.7 cells. Food Chem. Toxicol..

[43] Fotakis G, Timbrell JA (2006). *In vitro* cytotoxicity assays: comparison of LDH, neutral red, MTT and protein assay in hepatoma cell lines following exposure to cadmium chloride. Toxicology Lett.

[44] Rampersad SN (2012). Multiple applications of Alamar Blue as an indicator of metabolic function and cellular health in cell viability bioassays. Sensors.

[45] Zheng GQ, Kenney PM, Lam LK (1992). Anethofuran, carvone, and limonene: potential cancer chemopreventive agents from dill weed oil and caraway oil. Planta Med..

[46] Talalay P, De Long MJ, Prochaska HJ (1988). Identification of a common chemical signal regulating the induction of enzymes that protect against chemical carcinogenesis. Proc. Natl. Acad. Sci. U. S. A..

[47] Liu-Bryan R, Terkeltaub R (2015). Emerging regulators of the inflammatory process in osteoarthritis. Nat. Rev. Rheumatol.

[48] Eitner A, Hofmann GO, Schaible HG (2017). Mechanisms of Osteoarthritic Pain. Studies in Humans and Experimental Models. Front. Mol. Neurosci..

[49] Takano S (2017). Nerve growth factor regulation and production by macrophages in osteoarthritic synovium. Clin. Exp. Immunol..

[50] Prabst K, Engelhardt H, Ringgeler S, Hubner H (2017). Basic Colorimetric Proliferation Assays: MTT, WST, and Resazurin. Methods Mol. Biol..

[51] Green LC (1982). Analysis of nitrate, nitrite, and [15N]nitrate in biological fluids. Anal. Biochem..

[52] Rosa SC (2009). Impaired glucose transporter-1 degradation and increased glucose transport and oxidative stress in response to high glucose in chondrocytes from osteoarthritic versus normal human cartilage. Arthritis Res. Ther..

[53] Pfaffl MW (2001). A new mathematical model for relative quantification in real-time RT-PCR. Nucleic Acids Res..

[54] Sousa C, Ribeiro M, Rufino AT, Leitao AJ, Mendes AF (2017). Assessment of cell line competence for studies of pharmacological GPR30 modulation. J. Recept. Signal Transduct. Res..

