# Supplementary material for: Standardised comparison of limonene-derived monoterpenes identifies structural determinants of anti-inflammatory activity
Source: Sci Rep. 2020 Apr 29;10:7199. doi: 10.1038/s41598-020-64032-1 (PMC7190660; doi:10.1038/s41598-020-64032-1)
Supplement: Supplementary file 1 — Supplementary information. [file 41598_2020_64032_MOESM1_ESM.pdf]

Supplementary material to:

Standardised comparison of limonene-derived monoterpenes identifies structural determinants of anti-inflammatory activity

Cátia Sousa[a,b,c], Alcino Jorge Leitão[a,b,c], Bruno Miguel Neves[d], Fernando Judas[a,c,e], Carlos Cavaleiro[b,f], Alexandrina Ferreira Mendes[a,b,c] *

Affiliations:

[a]Center for Neuroscience and Cell Biology, University of Coimbra, Coimbra, Portugal

[b]Faculty of Pharmacy, University of Coimbra, Coimbra, Portugal

[c]Centre for Innovative Biomedicine and Biotechnology, University of Coimbra, Coimbra, Portugal

[d]Department of Medical Sciences and Institute of Biomedicine – iBiMED, University of Aveiro, Aveiro, Portugal

[e]Orthopaedics Department and Bone Bank, University and Hospital Center of Coimbra, Coimbra, Portugal

[f]Chemical Process Engineering and Forest Products Research Centre, Chemical Engineering Department, Faculty of Sciences and Technology, University of Coimbra, Coimbra, Portugal

Figure S1

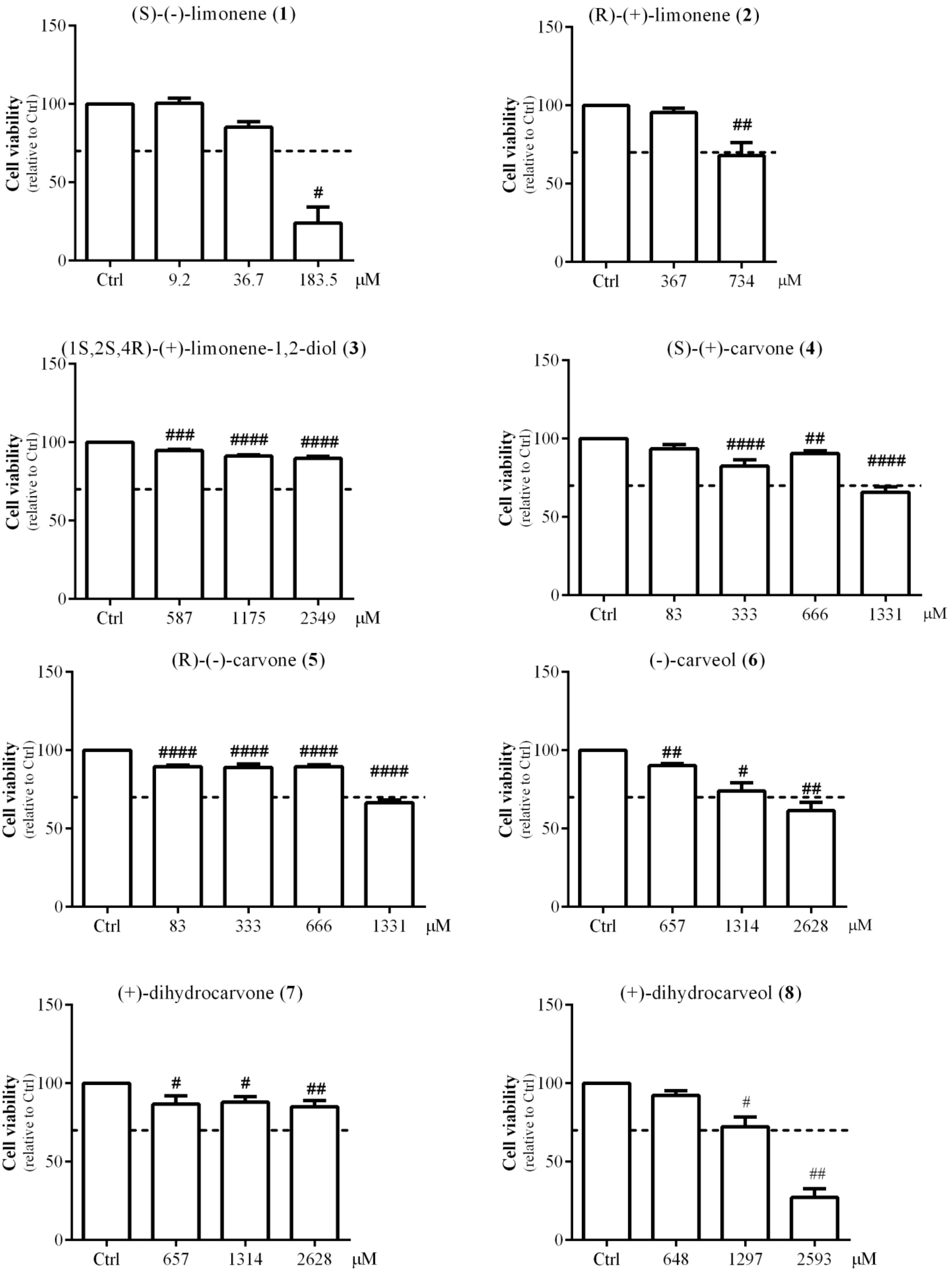

(S)-(-)-limonene (1)
Cell viability
(relative to Ctrl)
150
100
50
0
#
Ctrl
9.2
36.7
183.5
μM
(R)-(+)-limonene (2)
##
Ctrl
367
734
μM
(1S,2S,4R)-(+)-limonene-1,2-diol (3)
###
####
####
Ctrl
587
1175
2349
μM
(S)-(+)-carvone (4)
####
##
####
Ctrl
83
333
666
1331
μM
(R)-(-)-carvone (5)
####
####
####
####
Ctrl
83
333
666
1331
μM
(-)-carveol (6)
##
#
##
Ctrl
657
1314
2628
μM
(+)-dihydrocarvone (7)
#
#
##
Ctrl
657
1314
2628
μM
(+)-dihydrocarveol (8)
#
##
Ctrl
648
1297
2593
μM

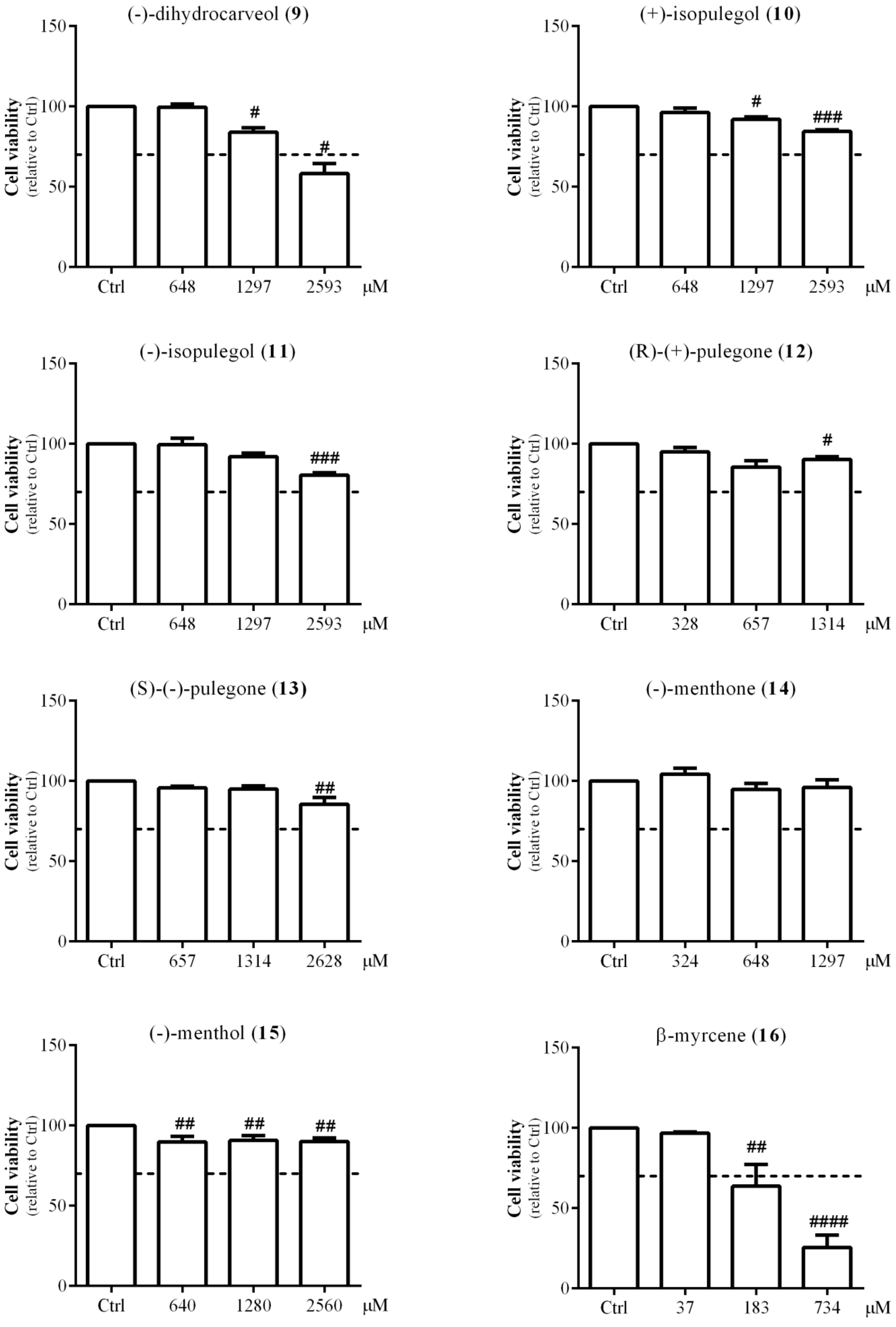

(-)-dihydrocarveol (9)
Cell viability (relative to Ctrl)
150
100
50
0
#
#
Ctrl
648
1297
2593
μM
(+)-isopulegol (10)
Cell viability (relative to Ctrl)
#
###
Ctrl
648
1297
2593
μM
(-)-isopulegol (11)
Cell viability (relative to Ctrl)
###
Ctrl
648
1297
2593
μM
(R)-(+)-pulegone (12)
Cell viability (relative to Ctrl)
#
Ctrl
328
657
1314
μM
(S)-(-)-pulegone (13)
Cell viability (relative to Ctrl)
##
Ctrl
657
1314
2628
μM
(-)-menthone (14)
Cell viability (relative to Ctrl)
Ctrl
324
648
1297
μM
(-)-menthol (15)
Cell viability (relative to Ctrl)
##
##
##
Ctrl
640
1280
2560
μM
β-myrcene (16)
Cell viability (relative to Ctrl)
##
####
Ctrl
37
183
734
μM

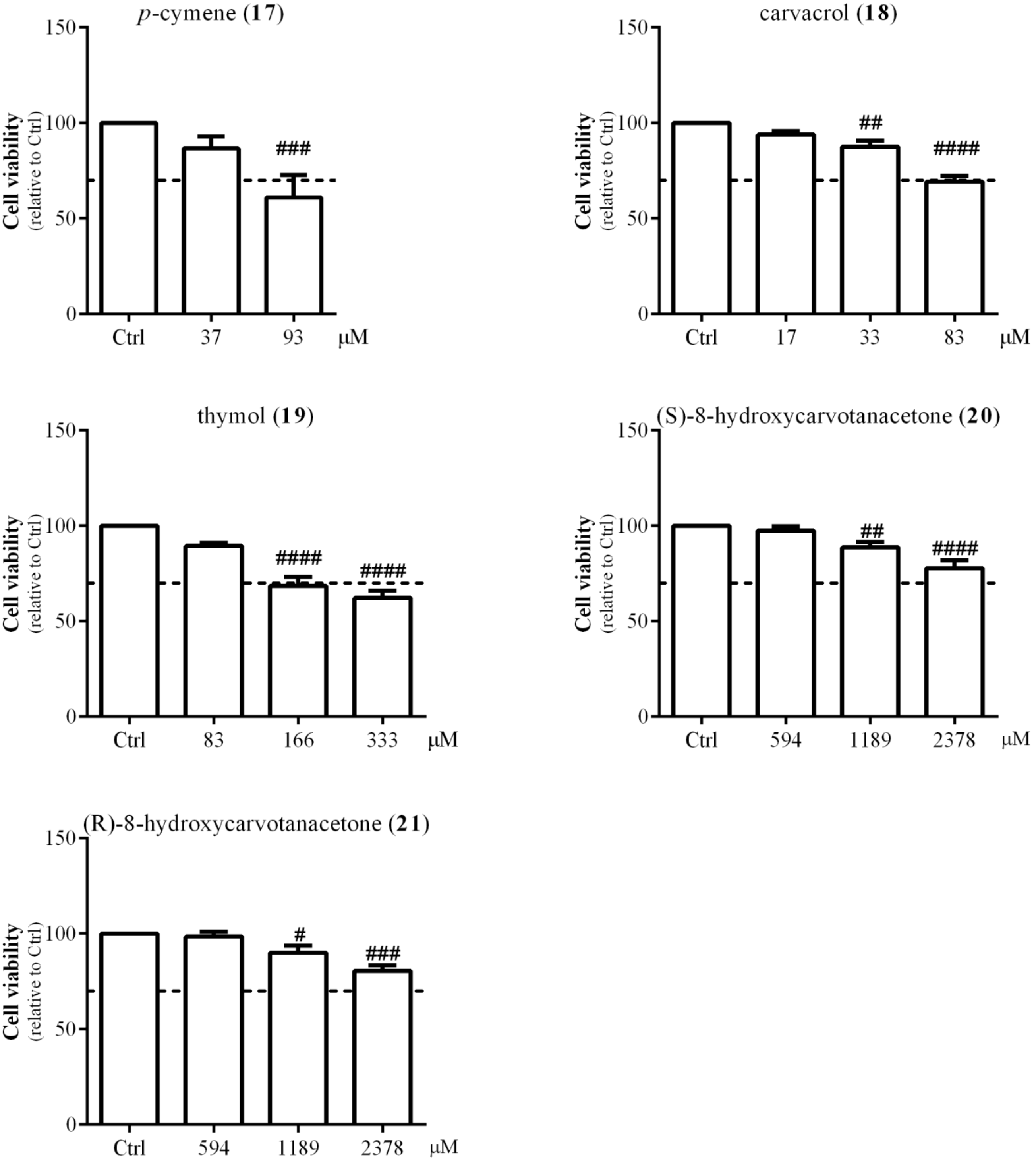

Figure S1. Evaluation of the cytotoxicity of the test compounds. Raw 264.7 cells were treated with the indicated concentrations of the each compound or with the vehicle (0.1% DMSO, control, Ctrl) for 18 h. Each column represents the mean ± SEM of, at least, three independent experiments. $^{\#}p \leq 0.05$, $^{\#\#}p \leq 0.01$, $^{\#\#\#}p \leq 0.001$ and $^{\#\#\#\#}p \leq 0.0001$ relative to Ctrl. The dotted line represents the threshold (70% of maximal viability) below which cytotoxicity is recognized, in agreement with standard ISO 10993-5.

Figure S2

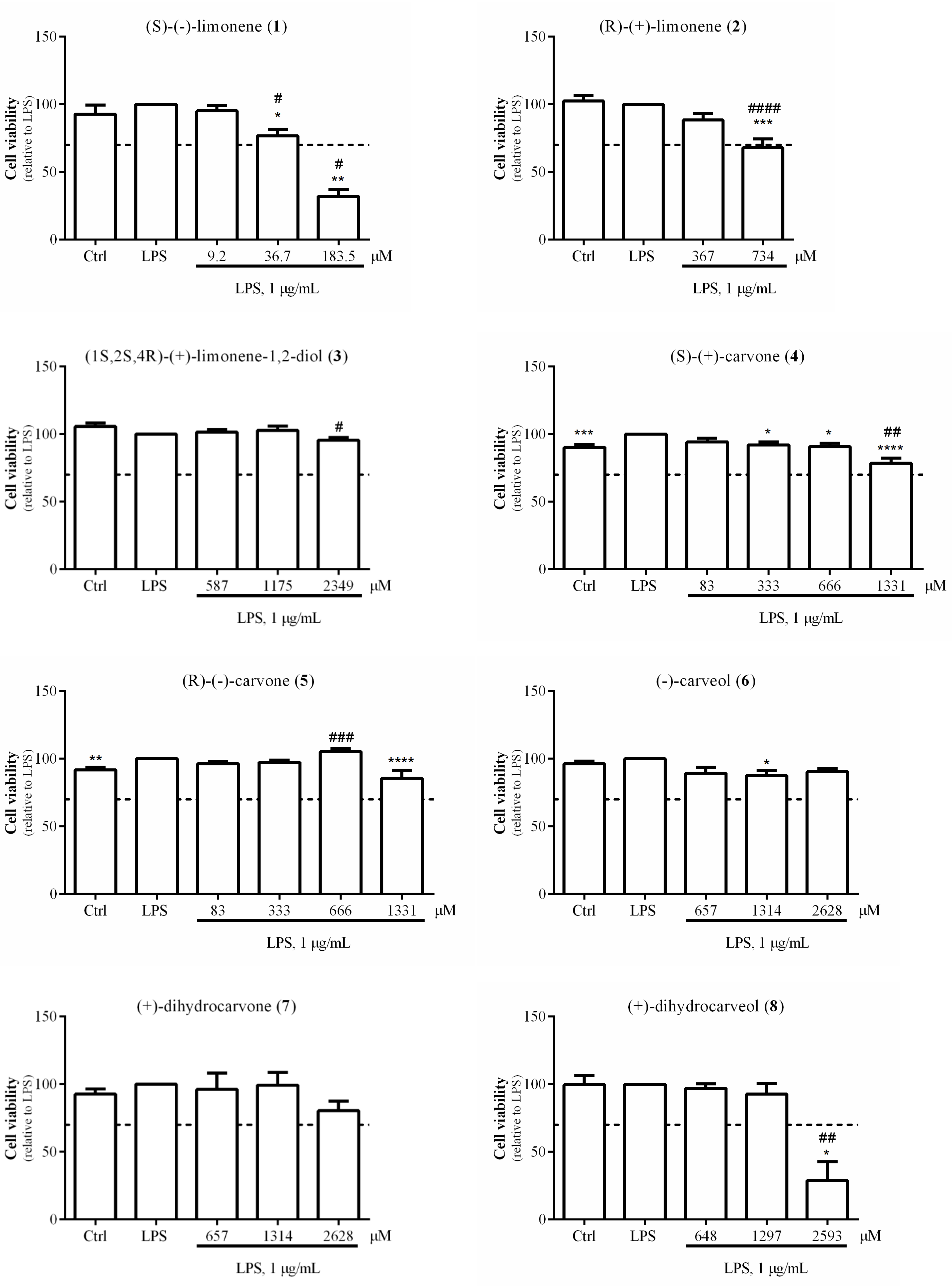

(S)-(-)-limonene (1)
(R)-(+)-limonene (2)
(1S,2S,4R)-(+)-limonene-1,2-diol (3)
(S)-(+)-carvone (4)
(R)-(-)-carvone (5)
(-)-carveol (6)
(+)-dihydrocarvone (7)
(+)-dihydrocarveol (8)
Cell viability
(relative to LPS)
0
50
100
150
Ctrl
LPS
9.2
36.7
183.5
367
734
587
1175
2349
83
333
666
1331
657
1314
2628
648
1297
2593
μM
LPS, 1 μg/mL
#
*
**
***
****
##
###
####

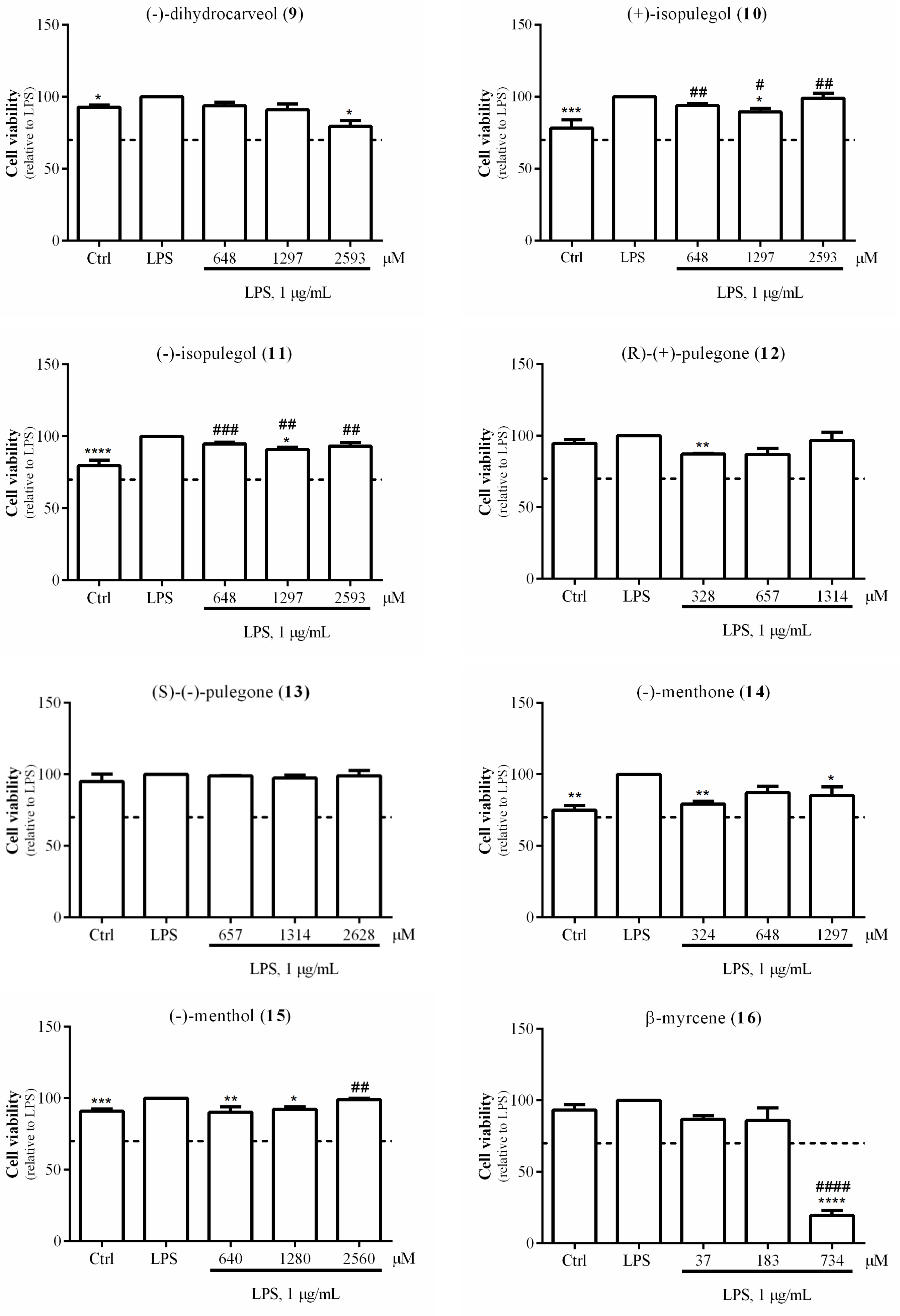

(-)-dihydrocarveol (9)
(+)-isopulegol (10)
(-)-isopulegol (11)
(R)-(+)-pulegone (12)
(S)-(-)-pulegone (13)
(-)-menthone (14)
(-)-menthol (15)
β-myrcene (16)
Cell viability
(relative to LPS)
0
50
100
150
Ctrl
LPS
648
1297
2593
324
328
657
1314
2628
640
1280
2560
37
183
734
μM
LPS, 1 μg/mL
*
**
***
****
#
##
###
####

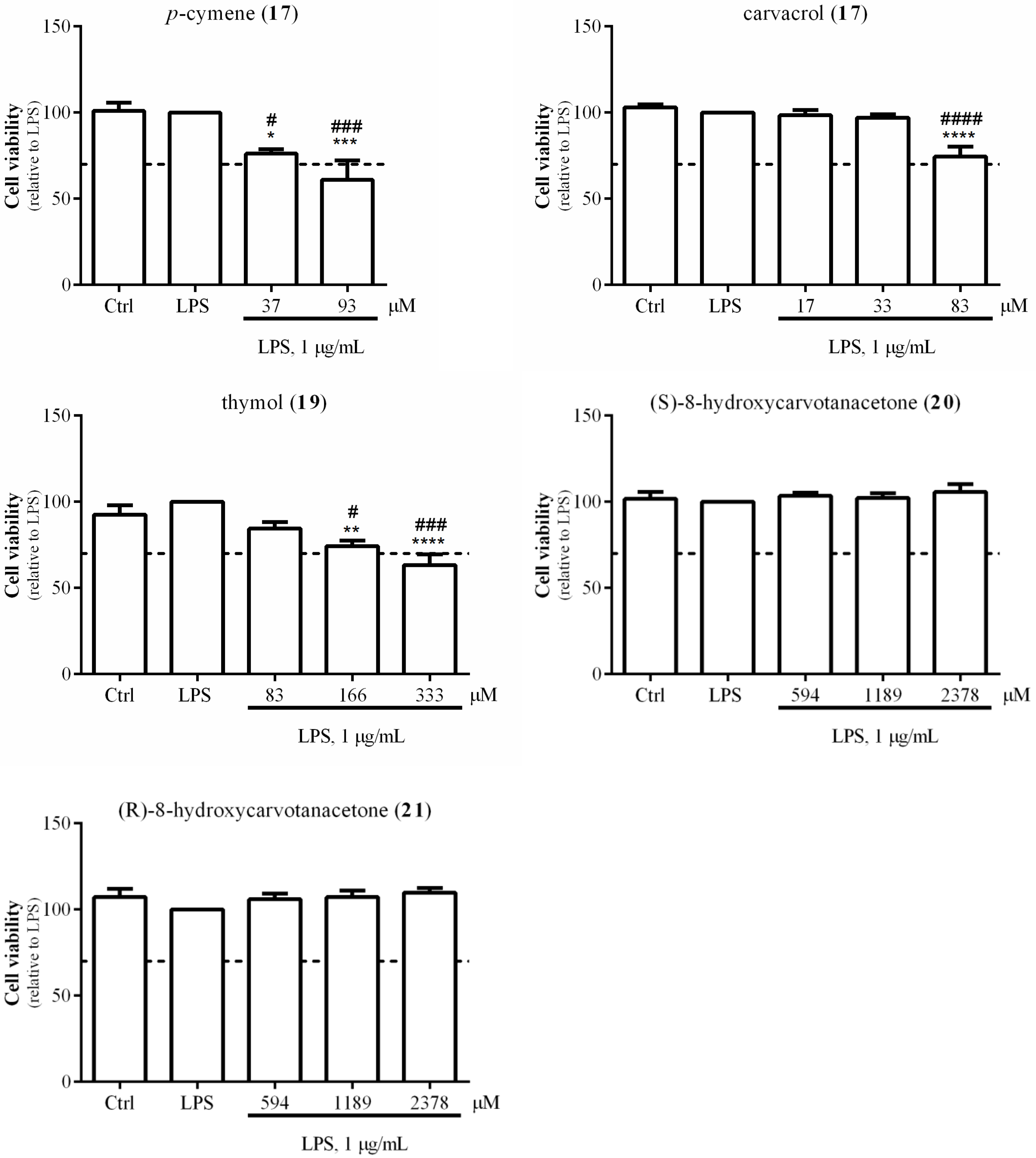

Figure S2. Evaluation of the cytotoxicity of the test compounds in the presence of bacterial lipopolysaccharide (LPS). Raw 264.7 cells were treated with LPS, 1 μg/mL, for 18 h, following pre-treatment for 1 h with the indicated concentrations of the test compounds or with vehicle (0.1% DMSO, Ctrl). Each column represents the mean ± SEM of ,at least, three independent experiments. *p ≤ 0.05, **p ≤ 0.01, ***p ≤ 0.001 and ****p ≤ 0.0001 relative to LPS-treated cells. $^{\#}$p ≤ 0.05, $^{\#\#}$p ≤ 0.01, $^{\#\#\#}$p ≤ 0.001 and $^{\#\#\#\#}$p ≤ 0.0001 relative to Ctrl. The dotted line represents the threshold (70% of maximal viability) below which cytotoxicity is recognized, in agreement with standard ISO 10993-5.

Figure S3

Uncropped blots shown in **Figure 2b**

(S)-(+)-carvone (**4**)

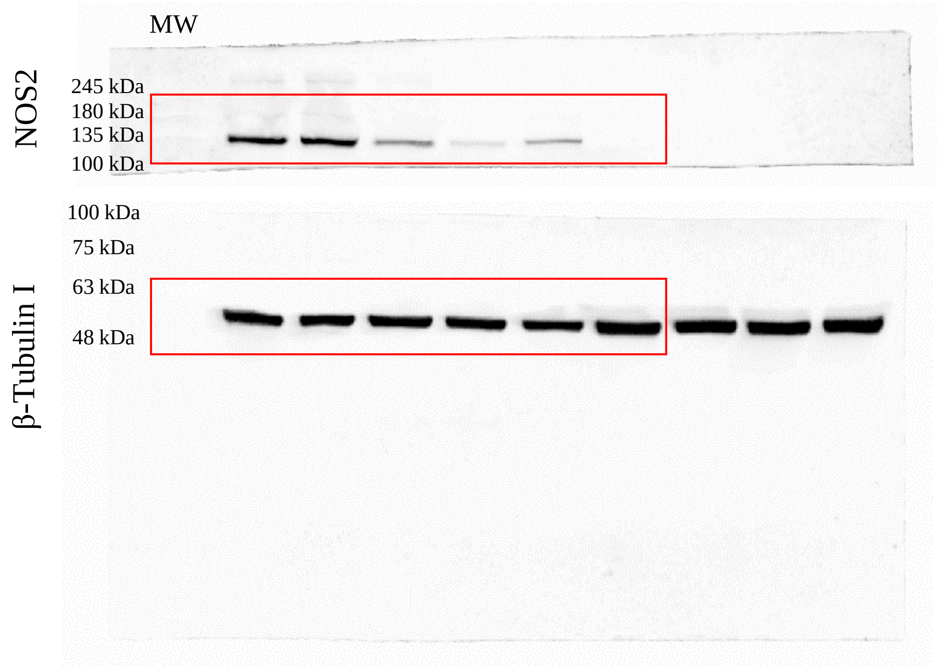

(R)-(-)-carvone (**5**)

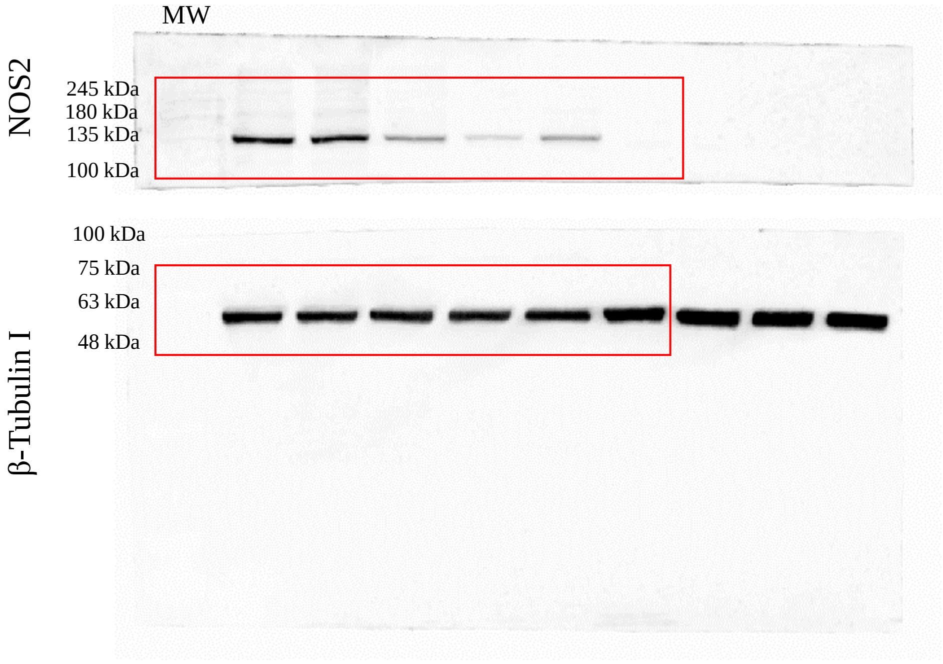

The membranes were cut at ≈ 100 kDa so that the upper piece was incubated with anti-NOS2 antibody and the lower one with the anti-β-Tubulin I antibody.

Uncropped blots shown in **Figure 3b**

(S)-(+)-carvone (**4**)

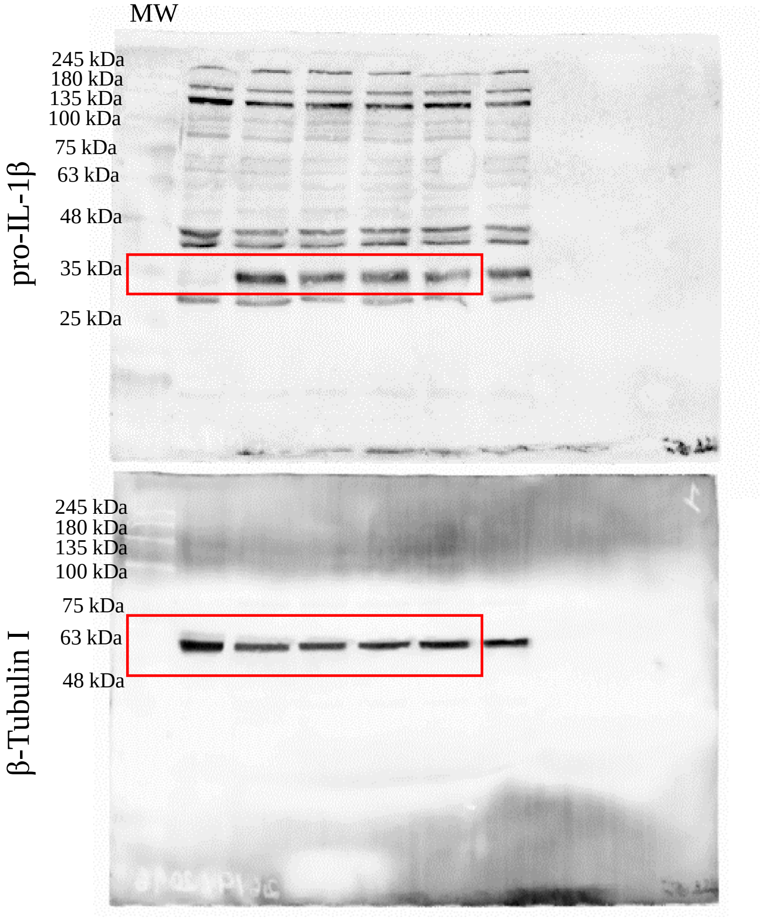

(R)-(-)-carvone (**5**)

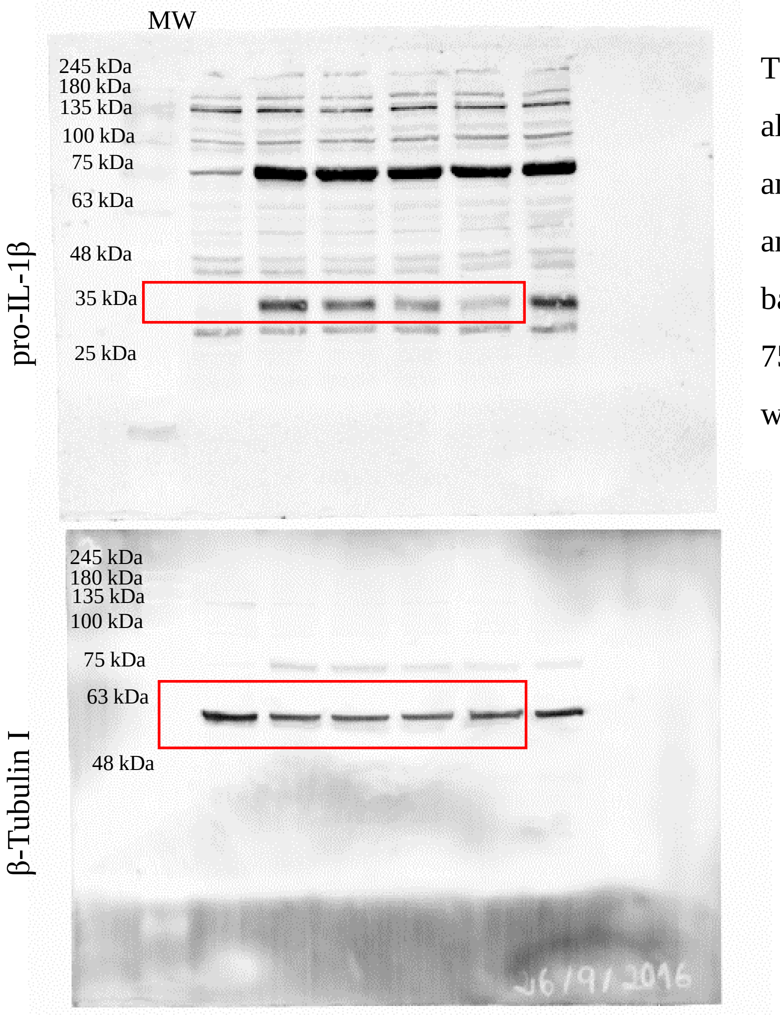

This membrane was also incubated with anti-COX2 antibody and the corresponding band appears near the 75 kDa molecular weight marker.

Uncropped blots shown in **Figure 4b**

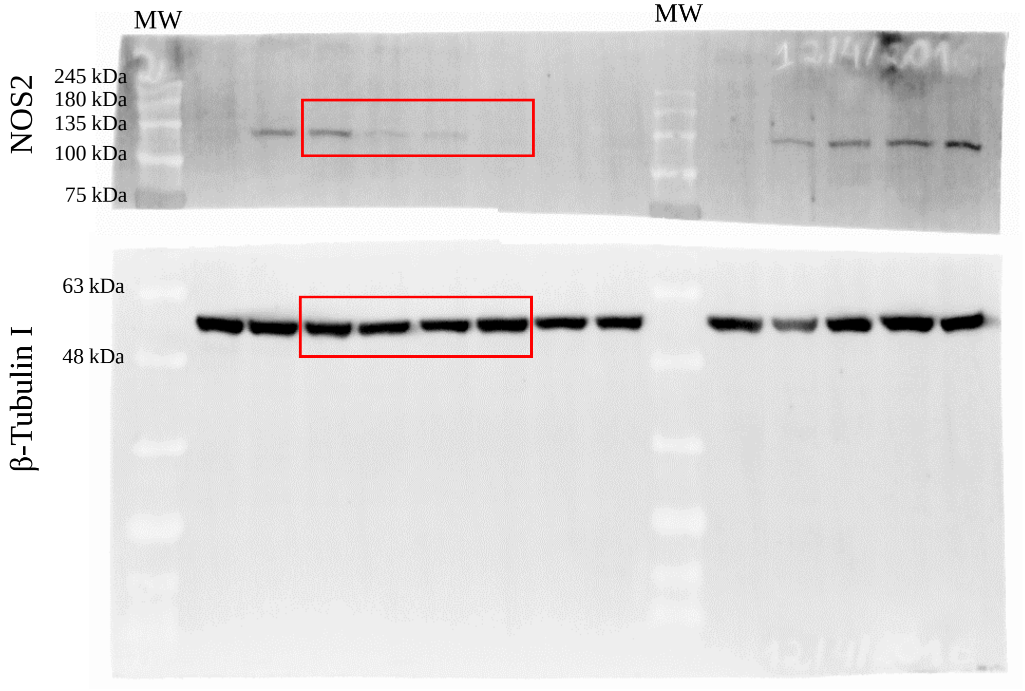

Uncropped blots shown in **Figure 4c**

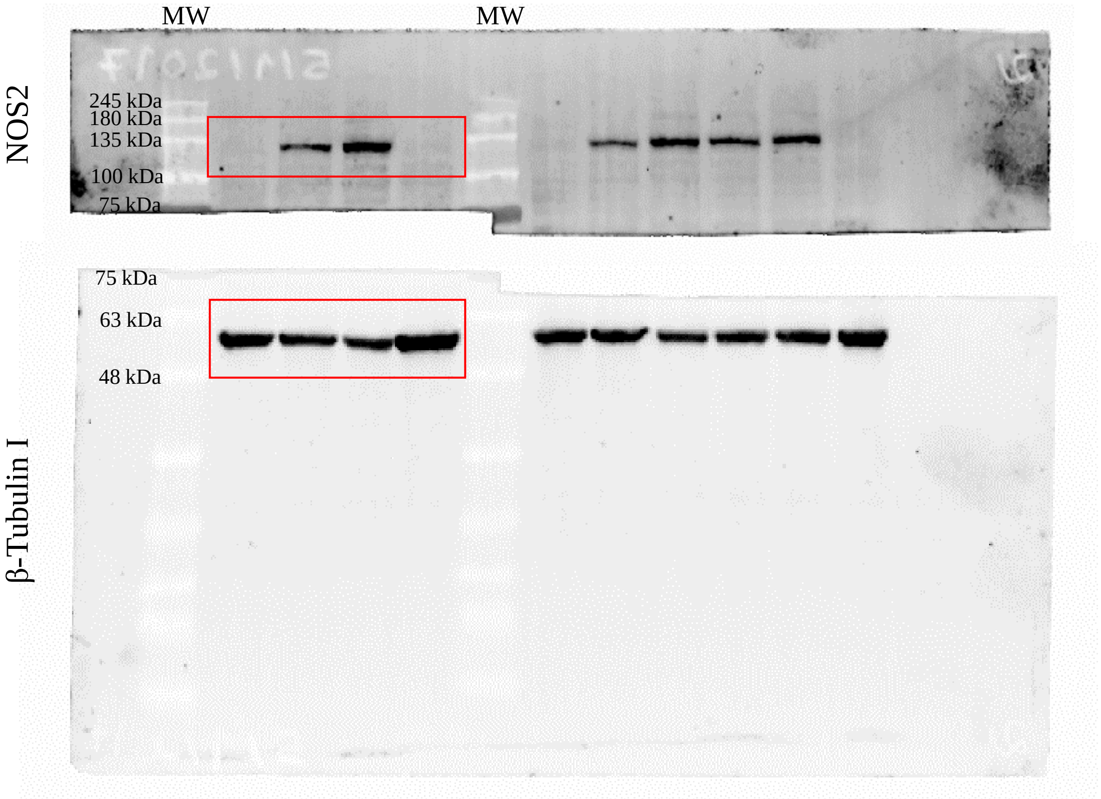

The membranes were cut at ≈ 75 kDa so that the upper piece was incubated with anti-NOS2 antibody and the lower one with the anti-β-Tubulin I antibody.

Uncropped blots shown in **Figures 5b and 5d**

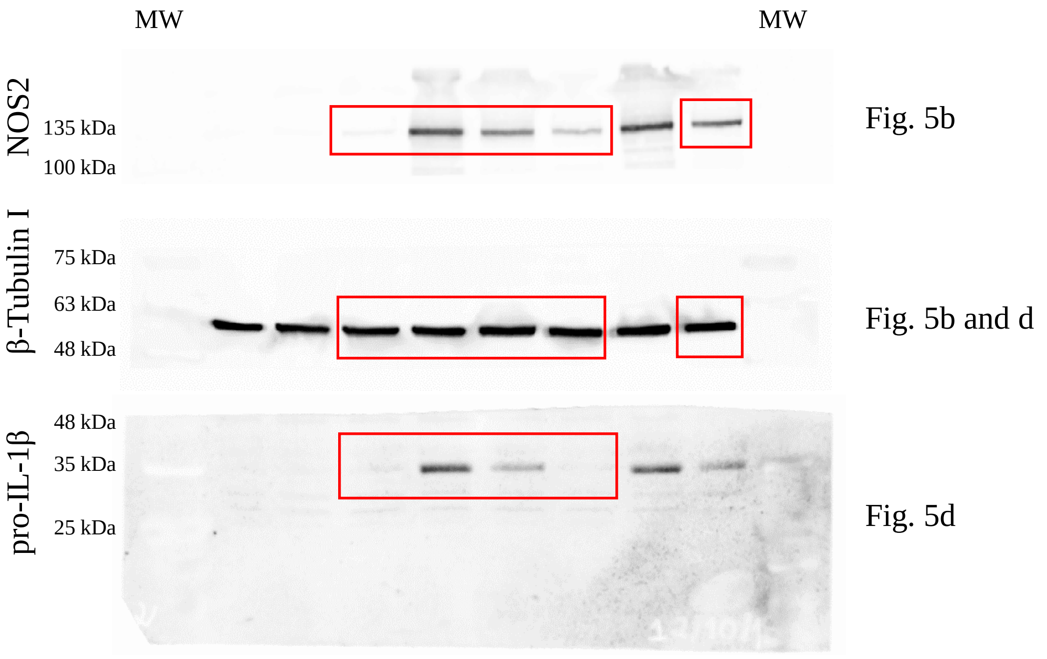

The membrane was cut between 100-75 kDa and 48 kDa so that the upper piece was incubated with anti-NOS2 antibody, the middle piece with anti-β-Tubulin I antibody and the lower one with the anti-IL-1β antibody.

Figure S3: Uncropped images of the blots shown in figures 2-5.

Table S1. Purity and isomer composition of the test compounds

Nº	Trivial name	IUPAC name of major isomer	Purity (%)*	Isomer composition	Catalog #, Vendor
**1**	(S)-(-)-limonene	(4S)-1-methyl-4-prop-1-en-2-ylclyclohexene	≥ 95%**	Purum	#62130, Sigma-Aldrich Co.
**2**	(R)-(+)-limonene	(4R)-1-methyl-4-prop-1-en-2-yl-cyclohexene	≥ 99%	Purum	#62118, Sigma-Aldrich Co.
**3**	(1S,2S,4R)-(+)-limonene-1,2-diol	(1S,2S,4R)-1-methyl-4-prop-1-en-2-ylcyclohexane-1,2-diol	≥ 97%	Purum	#669768, Sigma-Aldrich Co.
**4**	(S)-(+)-carvone	(5S)-2-methyl-4-prop-1-en-2-ylcyclohex-2-en-1-one	≥ 96%**	Purum	#435759, Sigma-Aldrich Co.
**5**	(R)-(-)-carvone	(5R)-2-methyl-4-prop-1-en-2-ylcyclohex-2-en-1-one	98%	Purum	#124931, Sigma-Aldrich Co.
**6**	(-)-carveol	(1R,5R)-2-methyl-5-prop-1-en-2-ylcyclohex-2-en-1-ol (1S,5R)-2-methyl-5-prop-1-en-2-ylcyclohex-2-en-1-ol	≥ 98%	Mixture of isomers	#61370, Sigma-Aldrich Co.
**7**	(+)-dihydrocarvone	(2R,5R)-2-methyl-5-prop-1-en-2-ylcyclohexan-1-one (2S,5R)-2-methyl-5-prop-1-en-2-ylcyclohexan-1-one	≥ 98%	Mixture of isomers	#09164, Sigma-Aldrich Co.
**8**	(+)-dihydrocarveol	(1S,2S,5S)- 2-methyl-5-prop-1-en-2-ylcyclohexan-1-ol	≥ 95%	Mixture of isomers – composition: n, ~ 75% iso, ~ 6% neo, ~ 3% neoiso, ~ 1.3%	#37277, Sigma-Aldrich Co.
**9**	(-)-dihydrocarveol	(1R,2R,5R)- 2-methyl-5-prop-1-en-2-ylcyclohexan-1-ol	≥ 95%	Mixture of isomers – composition: n, ~ 75% iso, ~ 6% neo, ~ 3% neoiso, ~ 1.3%	#37278, Sigma-Aldrich Co.

**10**	(+)-isopulegol	(1S,2R,5S)-5-methyl-2-prop-1-en-2-ylcyclohexan-1-ol	≥ 99%	Purum	#59765, Sigma-Aldrich Co.
**11**	(-)-isopulegol	(1R,2S,5R)-5-methyl-2-prop-1-en-2-ylcyclohexan-1-ol	≥ 99%	Purum	#59770, Sigma-Aldrich Co.
**12**	(R)-(+)-pulegone	(5R)-5-methyl-2-propan-2-ylidienecyclohexan-1-one	≥ 98.5%	Purum	#82569, Sigma-Aldrich Co.
**13**	(S)-(-)-pulegone	(5S)-5-methyl-2-propan-2-ylidienecyclohexan-1-one	98%	Purum	#328847, Sigma-Aldrich Co.
**14**	(-)-menthone	(2S,5R)-5-methyl-2-propan-2-ylcyclohexan-1-one	≥ 99%	Purum	#63677, Sigma-Aldrich Co.
**15**	(-)-menthol	(1R,2S,5R)-5-methyl-2-propan-2-ylcyclohexan-1-ol	≥ 99%	Purum	#63660, Sigma-Aldrich Co.
**16**	β-myrcene	7-methyl-3-methylideneocta-1,6-diene	≥ 90%	Purum	#64643, Sigma-Aldrich Co.
**17**	*p*-cymene	1-methyl-4-propan-2ylbenzene	≥ 99.5%	Purum	#30039, Sigma-Aldrich Co.
**18**	carvacrol	2-methyl-5-propan-2ylphenol	≥ 97%	Purum	#22051, Sigma-Aldrich Co.
**19**	thymol	5-methyl-2-propan-2ylphenol	98%	Purum	#30433, BDH

*Purity relative to sum of enantiomers determined by gas chromatography

The purities of (1**) (96.9%) and (**4**) (96.8%), which were in stock at our lab for over a year, were confirmed by GC-MS prior to starting the experiments and found to be within the limits defined by the manufacturer.
